# Activation of the Immune-Metabolic Receptor GPR84 Enhances Inflammation and Phagocytosis in Macrophages

**DOI:** 10.3389/fimmu.2018.01419

**Published:** 2018-06-20

**Authors:** Carlota Recio, Daniel Lucy, Gareth S. D. Purvis, Poppy Iveson, Lynda Zeboudj, Asif J. Iqbal, Daniel Lin, Chris O’Callaghan, Lucy Davison, Esther Griesbach, Angela J. Russell, Graham M. Wynne, Lea Dib, Claudia Monaco, David R. Greaves

**Affiliations:** ^1^Sir William Dunn School of Pathology, University of Oxford, Oxford, United Kingdom; ^2^Department of Chemistry, University of Oxford, Oxford, United Kingdom; ^3^Nuffield Department of Medicine, Wellcome Trust Centre for Human Genetics, University of Oxford, Oxford, United Kingdom; ^4^Department of Pharmacology, University of Oxford, Oxford, United Kingdom; ^5^Kennedy Institute of Rheumatology, University of Oxford, Oxford, United Kingdom

**Keywords:** immunometabolism, inflammation, metabolic G protein-coupled receptors, GPR84, macrophages

## Abstract

GPR84 is a member of the metabolic G protein-coupled receptor family, and its expression has been described predominantly in immune cells. GPR84 activation is involved in the inflammatory response, but the mechanisms by which it modulates inflammation have been incompletely described. In this study, we investigated GPR84 expression, activation, and function in macrophages to establish the role of the receptor during the inflammatory response. We observed that GPR84 expression in murine tissues is increased by endotoxemia, hyperglycemia, and hypercholesterolemia. *Ex vivo* studies revealed that GPR84 mRNA expression is increased by LPS and other pro-inflammatory molecules in different murine and human macrophage populations. Likewise, high glucose concentrations and the presence of oxidized LDL increased GPR84 expression in macrophages. Activation of the GPR84 receptor with a selective agonist, 6-(octylamino) pyrimidine-2,4(1H,3H)-dione (6-*n*-octylaminouracil, 6-OAU), enhanced the expression of phosphorylated Akt, p-ERK, and p65 nuclear translocation under inflammatory conditions and elevated the expression levels of the inflammatory mediators TNFα, IL-6, IL-12B, CCL2, CCL5, and CXCL1. In addition, GPR84 activation triggered increased bacterial adhesion and phagocytosis in macrophages. The enhanced inflammatory response mediated by 6-OAU was not observed in GPR84^−/−^ cells nor in macrophages treated with a selective GPR84 antagonist. Collectively, our results reveal that GPR84 functions as an enhancer of inflammatory signaling in macrophages once inflammation is established. Therefore, molecules that antagonize the GPR84 receptor may be potential therapeutic tools in inflammatory and metabolic diseases.

## Introduction

The concept of immunometabolism was first described in 2011, and it has emerged as a new field of scientific study (1). It highlights the regulatory interactions between innate and adaptive immunity and metabolism and provides new insights into disease mechanisms with the potential to identify new targets for therapeutic intervention (2, 3).

Macrophages are innate immune cells that play a pivotal role during the inflammatory response, acting as cellular mediators of both acute and chronic inflammation (4). Macrophages can be of bone marrow origin or can be tissue-resident macrophages derived from fetal monocytes (5). Their major functions include phagocytosis, antigen presentation, and immunomodulation through the production of cytokines, chemokines, and other inflammatory mediators. They are critical cells in the initiation, maintenance, and resolution of inflammation and hence are interesting to target in inflammatory disease (6).

Within the G protein-coupled receptor (GPCR) superfamily, a subset of metabolite-sensing GPCRs has been shown to actively participate in inflammation during metabolic disease. They have been reported to respond to intermediates of energy metabolism including free-fatty acids, lactate, succinate, and ketone bodies, among others (3, 7). GPCRs are seven transmembrane domain receptors that couple to heterotrimeric G proteins (Gαi, Gαs, Gαq, Gα_12/13_) and/or β-arrestins. These GPCRs regulate the activity of secondary-messenger producing enzymes or ion channels, modulating the intracellular concentrations of inositol triphosphate (IP3), diacylglycerol, cyclic AMP (cAMP), or calcium, among others. Consequently, they regulate the downstream intracellular signaling of kinase cascades, such as ERK/MAPK, JNK, p38, and Akt/PI3K. These pathways are central to the control of cell proliferation, differentiation, survival, migration, and other essential biological functions (3, 8, 9).

The GPR84 receptor was first identified in 2001, and it couples to the *Bordetella pertussis* toxin-sensitive Gαi protein, which inhibits adenylate cyclase activity (10, 11). Several studies have characterized GPR84 as a medium chain fatty acid (MCFA) receptor, with capric acid (a 10-carbon chain fatty acid) being the most potent ligand (11). However, GPR84 is not officially a “deorphanized” receptor, yet, as there is not a consensus that MCFAs are the major endogenous ligands that activate GPR84 (12). Numerous studies have focused their attention on developing new GPR84 agonist compounds with improved potency, selectivity, and specificity. 6-*n*-octylaminouracil 4 (6-OAU) was first reported by Suzuki et al. to be a novel GPR84-specific tool compound with far greater potency than hydroxylated or native MCFAs (EC_50_ values of ~500 nM for 6-OAU versus ~4 μM for capric acid in transfected GPR84-CHO cells) (13). Other small molecule screening programmes have identified further agonists that are active at the human GPR84 receptor expressed in transfected cell systems (14–16). However, these compounds have not been tested in relevant primary cell types.

A potential role for GPR84 in the regulation of inflammation has been suggested by several authors. It has previously been reported that the GPR84 agonist, diindolylmethane 2 (DIM), amplified IL-12 p40 mRNA expression in the LPS-stimulated RAW 264.7 macrophage cell line (11). Furthermore, GPR84 was shown to promote chemotaxis and pro-inflammatory cytokine release in human polymorphonuclear leukocytes and macrophages (13). In the context of metabolic disease, it has been suggested that GPR84 may influence lipid metabolism, as, compared to normal mice, GPR84-deficient mice exhibited smaller livers and increased triglyceride accumulation in response to FFA diet (17).

In this study, we aimed to identify the molecular and cellular responses following GPR84 activation in macrophages. We report that GPR84 expression is upregulated in macrophages by endotoxemia, hyperglycemia, and hypercholesterolemia. Therefore, we investigated intracellular signaling following GPR84 activation. We found that the synthetic GPR84 agonist 6-OAU upregulated the Akt, ERK, and the nuclear factor κB (NFκB) signaling pathways, amplified the expression of inflammatory mediators, and increased bacterial adhesion and phagocytosis in murine pro-inflammatory macrophages. None of these effects were observed in macrophages derived from GPR84 deficient mice or macrophages pretreated with a synthetic GPR84-specific antagonist.

## Materials and Methods

### Materials

All cell culture media, buffers, and other laboratory chemicals were obtained from Sigma-Aldrich (Gilligham, UK) unless otherwise specified. Pam3CysK and purified LPS were purchased from InVivogen (San Diego, CA, USA). Cytokines and chemokines were purchased from Peprotech (London, UK) and R&D Systems (Abingdon, UK). CIM-plate 16 and E-plate 96 PET were purchased from ACEA Biosciences (San Diego, CA, USA). pH-rodo Green *E. coli* Bioparticles conjugated for phagocytosis were obtained from ThermoFisher Scientific (Loughborough, UK). Rabbit anti-phospho-ERK (D13.14.4E), rabbit anti-total-ERK, rabbit anti-phospho-Akt (D9E), rabbit anti-total-Akt, β-actin, were purchased from Cell Signaling Technologies (Danvers, MA, USA). Mouse anti-p-65 was obtained from Santa Cruz Biotechnology (Dallas, TX, USA) and rabbit anti-GPR84 antibodies were from Santa Cruz Biotechnology and from Bioss Antibodies (Woburn, MA, USA). Rabbit anti-histone-H3 and mouse anti-tubulin were purchased from AbCam (Cambridge, UK). HRP-conjugated secondary antibodies and Bio-gel polyacrylamide beads were obtained from Biorad Laboratories (Hemel Hempstead, UK). Donkey anti-mouse Alexa Fluor 488 was obtained from ThermoFisher Scientific. Recombinant Mouse Complement Component C5a Protein was purchased from R&D Systems (Abingdon, UK). Pertussis toxin (PTX), a selective Gi-coupled signaling inhibitor, was purchased from ThermoFisher Scientific. 6-OAU was purchased from AstaTech (Bristol, PA, USA). The GPR84 antagonist, first described in a patent from Galapagos NV Company (US 2013/0165437 A1), was synthesized in-house as described in the supplementary methods. Both compounds were dissolved in 100% DMSO and then diluted in cell media to reach their respective working concentrations in 0.3% DMSO.

### Animals

All animal studies were conducted with ethical approval from the Dunn School of Pathology Local Ethical Review Committee and in accordance with the UK Home Office regulations (Guidance on the Operation of Animals, Scientific Procedures Act, 1986).

Male (7–10 weeks) C57BL/6 mice were obtained from the Biomedical Services Unit (Oxford, UK). Breeding pairs of non-obese diabetic (NOD) mice on a NOD/ShiLtJ background were originally obtained from Charles River (Calco, Italy), and their offspring were inter-crossed to provide mice for this experiment. Apolipoprotein E knockout (ApoE^−/−^) mice were purchased from Charles River. GPR84^tm1.1^ (KOMP) Vlcg sperm was obtained from the KOMP Repository at UC Davis. Heterozygous mice were re-derived through *in vitro* fertilization at Mary Lyon Center, MRC Harwell on a C57BL6/N background. Generated heterozygous mice were further bred to generate WT and Gpr84^−/−^ mice. All mice were housed in a 12-h light/12-h dark cycle unit with free access to food and water.

### *In Vivo* Studies and Tissue Collection

#### Protocol I. Endotoxemia Model

Male C57BL/6 mice were injected intraperitoneally (i.p.) with 1 mg/kg LPS or PBS and were monitored until sacrifice 2 and 8 h post injection.

#### Protocol II. Diabetes Model

For diabetic mice experiments, the autoimmune type 1 diabetes model of NOD mice was used. Female 11- to 15-week-old mice were monitored weekly for glycosuria from 9 to 10 weeks of age until the onset of diabetes. Once they were detected as being diabetic by presence of glucose in the urine, animals were sacrificed and tissues were harvested for analysis. Non-diabetic NOD mice of same age were used as littermate controls for the study.

#### Protocol III. Atherosclerosis Model

For the *in vivo* hypercholesterolemia experiments, male 10-week-old ApoE^−/−^ mice were fed on a Western type high-fat diet [high-fat diet (HFD), 21% milk fat, 0.15% cholesterol, SDS Diets, Essex, UK] for 6 or 12 weeks and then sacrificed to harvest tissues. ApoE^−/−^ mice fed a regular chow diet were used as controls.

All animals were euthanized *via* asphyxiation with a rising concentration of CO_2_. The brain, fat pads, lungs, kidneys, colon, bone marrow, and aorta were harvested and weighed before RNA or protein isolation.

### Fluorescent-Activated Cell Sorting (FACS)

For determining which cell types were expressing high levels of *Gpr84* mRNA in the model of endotoxemia, bone marrow was harvested from PBS/LPS injected mice, and bone marrow cells were harvested. A single cell suspension was prepared followed by blockade of Fc receptors with a blocking antibody (2.4G2 BD Biosciences, UK). In the presence of the blocking antibody, cells were stained for surface markers CD11b (Clone M1/70 BioLengend), LyG6 (Clone 1A8 BD Biosciences, UK), and Ly6C (Clone HK1.4 BioLengend). Cell sorting was preformed using a BD FACSAria III (BD Biosciences, UK).

### Cell Culture

#### Murine Bone Marrow-Derived Macrophages (BMDMs)

Bone marrow-derived macrophages were generated as previously described (18). Briefly, fresh bone marrow cells from tibiae and femurs of male C57BL/6 and GPR84^−/−^ mice were isolated and cultured in Dulbecco’s Modified Eagle’s medium (DMEM) supplemented with 10% heat-inactivated fetal bovine serum (FBS), 10% L929 cell-conditioned media as a source of macrophage colony-stimulating factor (19), and 1% penicillin/streptomycin for 7 days. Bone marrow cells were seeded into 8 ml of medium in 100 mm non-tissue culture treated Petri dishes (Thermo Fisher Scientific, Sterilin, UK). On day 5, an additional 5 ml of medium was added. Gentle scrapping was used to lift cells off dish surface. BMDMs were then counted and resuspended in FBS-free media at the desired cell concentration for the different experiments.

#### Biogel Elicitation of Primary Mouse Macrophages

Adult male C57BL/6 mice were injected i.p. with 1 ml of sterile 2% bio-gel polyacrylamide beads (P100 fine 45–90 µm) suspended in PBS. Mice were sacrificed 4 days later, and the elicited cells were collected by peritoneal lavage with 10 ml ice-cold PBS/2 mM EDTA. Cells were pelleted by centrifugation, resuspended in DMEM 10% FBS, and plated for 2–4 h to allow macrophages to attach to the plate and then were treated for experiments. Cells after the seeding have been previously demonstrated to be highly enriched for macrophages (20).

#### Resident Peritoneal Macrophages

Male C57BL/6 mice were sacrificed and peritoneal cavities were lavaged with 10 ml ice-cold PBS supplemented with 2 mM EDTA. Cells were pelleted by centrifugation, resuspended in DMEM 10% FBS, and plated for 2–4 h to allow macrophages to attach to the plate.

#### Murine Microglia

Meninges were removed from brains of C57BL/6 mice at postnatal days 1–3. Trypsin 0.25% and DNase I were used for mincing the cerebral tissue, which was filtered and resuspended in DMEM supplemented with 10% FBS and 1% penicillin/streptomycin, and seeded in culture flasks. After 10–12 days in culture, detached microglia were collected and plated on 6-well culture dishes (21).

### Human Monocyte-Derived Macrophages (hMDMs)

Peripheral blood mononuclear cells (PBMCs) were purified from healthy human volunteers by density centrifugation of fresh peripheral blood over Ficoll-Paque PLUS (GE Healthcare Life Sciences, Piscataway, NJ, USA). Written consent was obtained from healthy individual (Ethical approval: South Central-Hampshire B, reference 13/SC/0392). The PBMC layer was carefully harvested and then washed repeatedly with PBS and centrifugation until the supernatant became clear. CD14^+^ monocytes were then isolated from the PBMCs by positive selection using magnetic beads conjugated to anti-CD14 antibody (Miltenyi Biotec, Bergisch Gladbach, Germany). Cells were maintained in RPMI 1640 medium supplemented with 10% FBS, 50 ng/ml macrophage colony-stimulating factor (M-CSF, eBioscience, San Diego, CA, USA), 50 U/ml penicillin, and 50 µg/ml streptomycin for 7 days.

### Cell Treatments

Cells were seeded onto tissue culture plastic in FBS-free media and allowed to adhere overnight at 37°C, 5% CO_2_. Cells were then treated with compounds at concentrations indicated in Table 1.

**Table 1:** Compounds and their final working concentrations used.

Compound	Working concentration
cLPS	100 ng/ml
pLPS	100 ng/ml
Pam3CysK	300 ng/ml
IFNγ	20 ng/ml
IL4	20 ng/ml
IL13	20 ng/ml
Zymosan	10 µg/ml
Poly I:C	10 µg/ml
Flagellin	500 ng/ml
oxLDL	50 µg/ml
C5a	10 nM
Pertussis toxin	200 ng/ml
6-OAU	1 µM
GPR84 Antagonist	10 µM

For the glucose shock experiments, BMDMs were grown in low glucose (5.5 mM) DMEM supplemented with 10% FBS, 10% L929 cell conditioned medium, and 1% penicillin/streptomycin for 7 days prior to stimulation with 25 mM d-glucose or l-glucose for 4 h.

### Intracellular cAMP Measurement

Intracellular cAMP levels were measured using the DiscoverX HitHunter^®^ Assay (Birmingham, UK) following the manufacturer’s protocol. Briefly, CHO-K1 cells stably expressing human GPR84 receptor were plated into a 1/2 area 96-well plate (15,000 cells/well) and incubated at 37°C, 5% CO_2_ for 24 h before the medium was removed and replaced with PBS. In agonist mode, the cells were simultaneously treated with 25 µM forskolin and 6-OAU or vehicle for 30 min at 37°C, 5% CO_2_. In antagonist mode, the cells were pretreated with the antagonist or vehicle for 15 min, before stimulation with forskolin and 6-OAU at the EC_80_ concentration for 30 min at 37°C, 5% CO_2_. Cell lysis and cAMP detection were performed as per the manufacturer’s protocol. Luminescence measurements were taken using a PHERAstar FS microplate reader (BMG Labtech, Aylesbury, UK).

### ECIS 96-Well Assay

Wells of a 96-well ACEA E-plate were filled with 50 µl of media (RPMI + 25 mM HEPES) and a background signal was measured using the ACEA RTCA-SP system. Afterward, BMDMs (with or without LPS 0.1 µg/ml) were added to each well (50 µl = 50,000 cells/well) and left for 16 h at 37°C, 5% CO_2_, with Cell Index (CI) measurements made every 15 min. Cells were then treated with: vehicle (DMSO 0.3%); C5a (10 nM); 6-OAU (0.1–100 μM); capric acid (5–500 μM); lauric acid (5–500 μM); and undecanoic acid (5–500 μM). CI measurements were taken every 10 s for 3 h after compound addition. Data are displayed as change in CI from the point of compound addition (Δ Cell Index).

### Chemotaxis Assay

Real-time chemotaxis experiments were performed as described previously using the ACEA xCELLigence RTCA-DP instrument (22). Briefly, vehicle (0.3% DMSO), 10 nM C5a, or 1 µM 6-OAU was added dissolved in media (RPMI + 25 mM HEPES) to the lower chamber of a CIM-16 plate (160 µl/well). The upper chamber was attached and upper wells were filled with 50 µl of media following equilibration for 30 min. Then, the plate was transferred into the RTCA-DP system and the background signal was measured. BMDMs were then placed into the upper wells (50 µl–1 × 10^5^ cells/well). CI measurements were taken every 5 s over 6 h. Chemotactic responses were assessed by quantification of the slope of the curve in the first 90 min and by the maximum CI minus minimum CI (Max–Min) values of the curve over the entire experiment. Pooled data are displayed as a fold change relative to cells migrating toward vehicle alone.

### Migration Scratch Assay

The scratch assay is an easy, low-cost, and well-developed method to measure cell migration *in vitro* (23). BMDMs at 90–95% confluency were treated with either vehicle (0.3% DMSO) or 1 µM 6-OAU for 1 h, followed by a scratch injury in the middle of the well using a plastic P10 Gilson pipette tip. Cells were then stimulated ± 10 nM C5a and immediately placed into the IncuCyte Zoom platform at 37°C, 5% CO_2_. Images were taken every 4 for 24 h, and cell migration was quantified by counting the number of cells that had invaded the scratch at 24 h, considering the reference area at time 0 h.

### Bacterial Adhesion Assay

*E. coli* strain DH5-α was cultured for 16 h in 5 ml LB broth at 37°C with shaking at 180 rpm, and a sub-culture was then prepared and grown for a further 3 h. Bacteria grown in exponential phase were adjusted to OD600 = 0.3 in DMEM containing 1% FBS (for DH5-α, a suspension at OD600 = 0.3 corresponds to 2.28e8 CFU/mL).

Medium with a bacterial suspension (MOI = 5) was added to BMDMs (1 × 10^5^ cells/well) previously treated with vehicle (DMSO 0.3%), 1 µM 6-OAU, or 10 µM antagonist + 1 µM 6-OAU. The cell plate was centrifuged at 300 *g* for 5 min, and then incubated for 30 min at 37°C before wells were washed with PBS three times. To measure bacteria attached to the macrophages, cells were lysed with 200 µl of 1% triton-X for 2 min, and then diluted by addition of 800 µl of PBS. Serially diluted cell lysates were plated on LB agar plates for 16 h, and colony forming units were counted.

### Phagocytosis Assay

#### IncuCyte Zoom Phagocytosis

Bone marrow-derived macrophages previously treated with either vehicle (0.3% DMSO) or 1 µM 6-OAU for 1 h were incubated with unopsonised pHrodo *E. coli* bioparticles at 0.1 mg/ml in a 96-well flat clear bottom plate. For the inhibition studies with a GPR84 antagonist, cells were pretreated with 10 µM antagonist for 30 min before addition of either vehicle or 6-OAU. The plate was then placed into the IncuCyte Zoom platform (Essen Bioscience, Welwyn Garden City, UK) which was housed inside a humidified incubator at 37°C, 5% CO_2_. Two to four images per well from three technical replicates were taken every 15 min for 4 h using a 20× objective lens and then analyzed using the IncuCyte™ Basic Software. Green channel acquisition time was 400 ms.

#### Bead Phagocytosis

Bone marrow-derived macrophagess previously treated with either vehicle (0.3% DMSO) or 1 µM 6-OAU for 1 h in 4-well chamber slides (Thermo Fisher Scientific) were incubated with opsonized 3 µm polystyrene micro particle beads (Sigma-Aldrich) for 1 h at 37°C, 5% CO_2_. Bead opsonization was performed preincubating the beads with human serum (1:10 dilution in bead solution) for 1 h at 37°C, 5% CO_2_. Cells were then washed with PBS and fixed with methanol followed by staining with safranin (Sigma-Aldrich). After several PBS washes, the chamber was disassembled and the slide was mounted with a cover slip using mounting media (Fluormount-G^®^, SouthernBiotech, Birmingham, AL, USA). Twenty images per well were taken using a Chromyx HD camera connected to ta Leica DME light microscope. Number of beads was manually counted by an observer blind to cell treatments.

### mRNA Expression Analysis

Mouse tissues and cultured cells were extracted with TRIzol reagent (Thermo Fisher Scientific), and total RNA concentration and quality was determined with a ND-1000 spectrophotometer (Nano Drop Technologies, Wilmington, DE, USA). cDNA was synthesized from 700 to 1,000 ng RNA using the QuantiTect Reverse Transcription kit (Qiagen, Manchester, UK) according to the manufacturer’s instructions. Real-time quantitative PCR was performed using either Taqman or Sybr Select gene expression master mix (Life Technologies) in the StepOnePlus™ thermal cycler (Applied Biosystems). Primers were purchased from Qiagen (*tnf*α, *il-6, il-12b, ccl2, ccl5, cxcl1, fc*γ*RI, icam-1, βactin*) and Taqman (*gpr84, βactin*). Cycle threshold values were determined by the StepOne software and target gene expression was normalized to housekeeping gene (*βactin*). Relative expression results were plotted as mRNA expression divided by actin expression, and normalized to basal samples when convenient (24).

### Protein Expression Analysis

#### Western Blot

Cells were lysed by adding RIPA buffer (Sigma-Aldrich) supplemented with protease and phosphatase inhibitors (Sigma-Aldrich) followed by manual disruption. BMDMs were separated into cytoplasmic and nuclear fractions following manufacturer’s protocol of the Subcellular Protein Fractionation kit from Thermo Scientific. Protein concentration was determined by using a BCA protein assay kit (Thermo Fisher Scientific). Total cell protein (20–30 mg) was added to 4× Laemmli buffer (250 mM Tris–HCl, pH 6.8, 8% SDS, 40% glycerol, 0.004% bromophenol blue, 20% b-mercaptoethanol) and heated at 95°C for 5 min. Samples were then resolved on SDS-PAGE gels and transferred onto Hybond ECL nitrocellulose membranes (GE Healthcare, Buckinghamshire, UK). Membranes were blocked with 5% milk in Tris-buffered saline with Tween (TBS-T) (Tris-buffered saline, 0.1% Tween-20) for 1 h at RT and then incubated with the primary antibody (rabbit anti-GPR84, rabbit anti-phospho-Akt, rabbit anti-phospho-ERK or mouse anti-p65) diluted 1:1,000 in 5% BSA/TBS-T overnight at 4°C. Next, membranes were incubated with an HRP-conjugated anti-rabbit secondary antibody for 1 h at RT. Protein bands were visualized by incubating the membranes for 5 min with Amersham ECL prime and subsequent exposure to X-ray film over a range of exposure times. β-actin, histone-H3, and tubulin were used as loading controls. For successive antibody incubations using the same membrane bound antibodies were removed with stripping buffer (Thermo Fisher Scientific). Full, uncropped blots can be found in Figure S7 in Supplementary Material, where also shown that similar results were obtained using total ERK and total Akt antibodies rather than β-actin for loading controls.

#### ELISA

Measurement of TNFα, IL6, and CCL2 secreted protein levels in cell supernatants was performed by ELISA (R&D Systems) according to manufacturer’s instructions.

#### Immunofluorescence

For p-65 expression and location detection by immunofluorescence, cells placed in a 4-well chamber-slide were fixed (4% paraformaldehyde) for 10 min at RT, permeabilized (0.5% Triton X-100 in PBS) for 10 min at 4°C, blocked with 4% BSA/PBS containing 6% donkey serum, and incubated with mouse anti-p-65 antibody diluted 1:100 in 4% BSA/PBS, overnight at 4°C. Cells were then incubated with donkey anti-mouse Alexa Fluor 488 diluted 1:500 in 4% BSA/PBS for 1 h at RT. DAPI (4’,6-diamidino-2-phenylindole) was used for nuclear counterstaining. The slide was mounted with Fluormount-G^®^ and cells were visualized using a confocal microscope.

### Statistical Analysis

All quantitative data are expressed as mean ± SEM of *n* independent biological replicates. Statistical significance was performed using a Student’s unpaired *t*-test, or one-way analysis of variance (ANOVA) followed by Dunnett’s multiple comparison *post hoc* test (Prism 7 GraphPad Software, San Diego, CA, USA). A *P* value of <0.05 was taken to be statistically significant.

## Results

### *Gpr84* Expression Is Increased During Acute Inflammation

**Figure 1:**
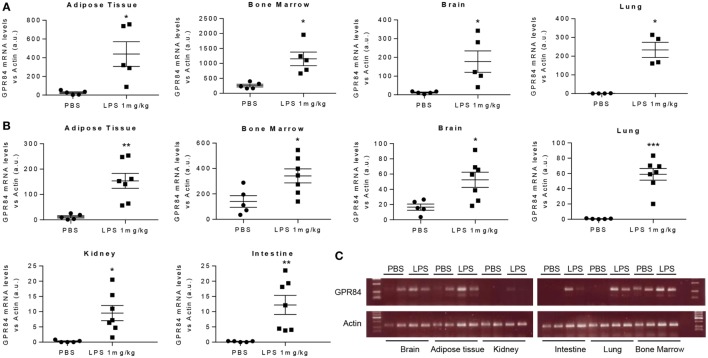
*Gpr84* expression is increased in mouse tissues in acute inflammation. C57BL/6 male mice were injected i.p. with 0.1 mg/kg of LPS or PBS (controls). Animals were sacrificed at 2 and 8 h and *Gpr84* expression was examined by q-PCR of the cDNA from mouse tissues. **(A)** *Gpr84* mRNA levels at 2 h post PBS or LPS injection in adipose tissue, bone marrow, brain, and lung. **(B)** *Gpr84* mRNA levels at 8 h post PBS or LPS injection in adipose tissue, bone marrow, brain, lung, kidney, and intestine. **(C)** Agarose gel electrophoresis of q-PCR products from mouse tissue cDNA of the 8 h endotoxemia experiment are shown. Products sizes are 139 bp for GPR84 and 72 bp for β-actin. Data presented as mean ± SEM. The number of animals is represented by dots in the graph. Statistical significance was assessed using a Student’s unpaired *t*-test. *P ≤ 0.05, **P ≤ 0.01, ***P ≤ 0.001 versus PBS control group.

To assess the pattern of *Gpr84* expression in mice during acute inflammation, we carried out an experimental model of endotoxemia. We administered 1 mg/kg LPS i.p. to male C57BL/6 mice and sacrificed at 2 and 8 h. As shown in Figure 1, LPS injection significantly increased *Gpr84* mRNA levels in mouse adipose tissue, bone marrow, brain, lung, kidney, and intestine compared to PBS-injected control mice, both at 2 (Figure 1A) and 8 h (Figures 1B,C). The highest levels of *Gpr84* mRNA were found in the bone marrow. As there are no commercially available specific antibodies against GPR84 (see Figure S1A in Supplementary Material), we were unable to confirm directly the *Gpr84* mRNA pattern of expression at protein level. In order to identify the main cell population responsible for the increased *Gpr84* expression, we performed FACS to sort monocytes, neutrophils, and total myeloid cells, excluding neutrophils/monocytes, from bone marrow isolated from mice treated with PBS or LPS. As shown in Figure S2 in Supplementary Material, only monocytes significantly increased *Gpr84* mRNA expression in the bone marrow of LPS-treated mice.

### M1 Pro-Inflammatory Macrophages Express High Levels of *Gpr84*

**Figure 2:**
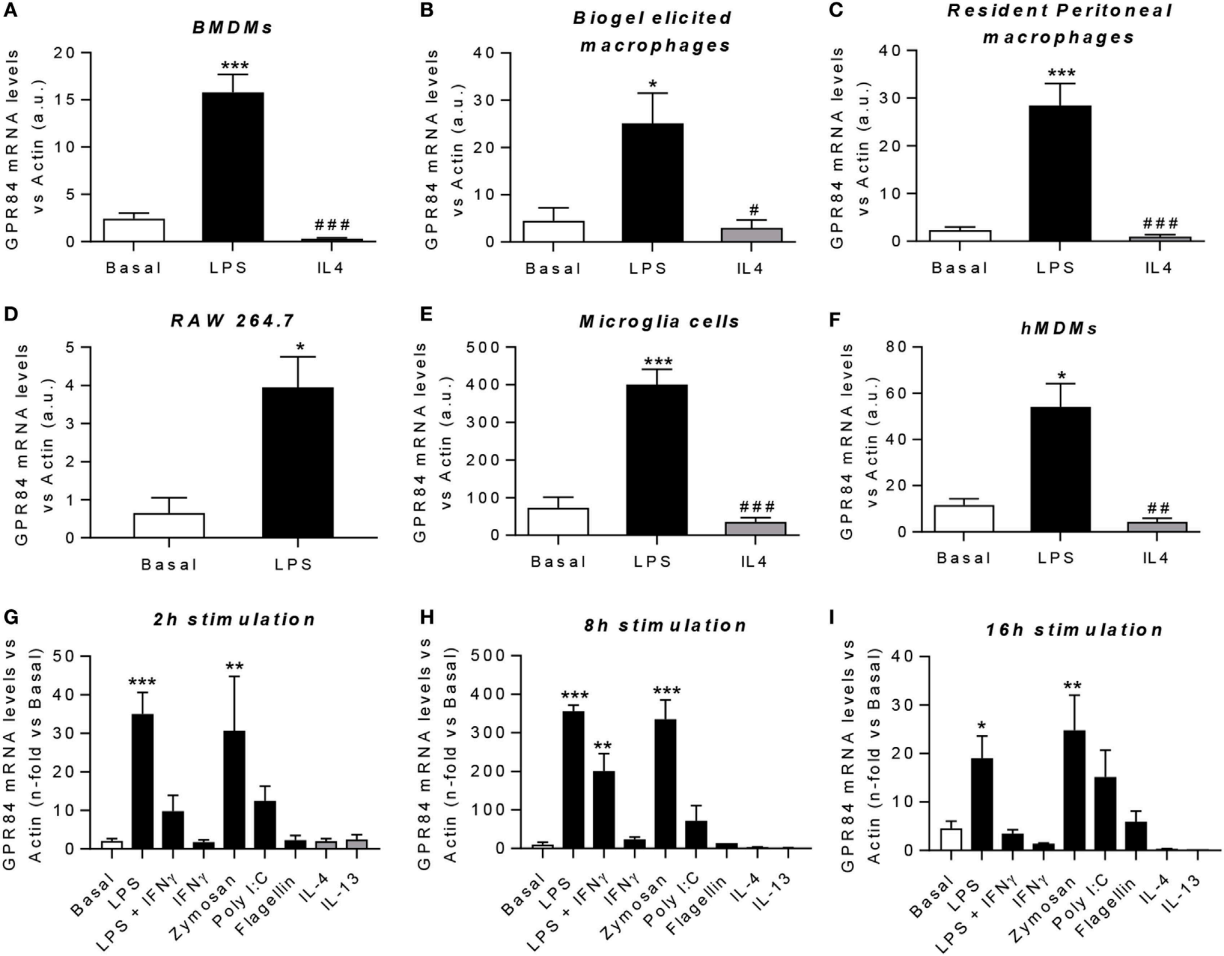
*Gpr84* expression is highly expressed by M1 pro-inflammatory macrophages. **(A–F)** mRNA expression of GPR84 was analyzed by q-PCR of cDNA prepared from bone marrow-derived macrophages (BMDMs) **(A)**, biogel-elicited macrophages **(B)**, resident peritoneal macrophages **(C)**, RAW 264.7 cells **(D)**, microglia cells **(E)**, and human monocyte-derived macrophages (hMDMs) **(F)** challenged with either LPS or IL-4 for 16 h. **(G–I)** mRNA expression of GPR84 receptor was analyzed by q-PCR on BMDMs following exposure to TLR ligands and cytokines for 2 **(G)**, 8 **(H)**, and 16 **(I)** h. Error bars represent SEM of *n* = 2–4 separate experiments. Statistical significance was assessed using one-way ANOVA with Dunnett’s multiple comparison *post hoc* test. *P ≤ 0.05, **P ≤ 0.01, ***P ≤ 0.001 versus basal, ^#^*P* ≤ 0.05, ^##^*P* ≤ 0.01, ^###^*P* ≤ 0.001 versus LPS.

Macrophages are key players during both acute and chronic inflammation. In order to investigate the expression of *Gpr84* in different human and murine macrophage populations, we first polarized cells into “M1-like” pro-inflammatory state or “M2-like” pro-resolution state. *Gpr84* mRNA was significantly upregulated by LPS in all murine macrophage cell populations, including BMDMs, biogel-elicited macrophages, resident peritoneal macrophages, the cell line RAW 264.7 and microglia (Figures 2A–E). Similar results were observed with hMDMs (Figure 2F). In contrast, *Gpr84* values were comparable to basal values in macrophages stimulated with IL-4. To further characterize macrophage *Gpr84* expression, we challenged BMDMs with a range of inflammatory stimuli including several pathogen-associated molecular patterns and selected cytokines for 2, 8, and 16 h (Figures 2G–I). Addition of LPS and zymosan resulted in increased BMDM expression of *Gpr84* at all time points, showing a significant peak at 8 h. In contrast, IL-4 and IL-13 did not stimulate *Gpr84* expression and, if anything, they downregulated receptor expression. Our results are in agreement with the published literature, indicating that GPR84 is strongly upregulated in macrophages under pro-inflammatory conditions (11, 25). All of the experiments reported in Figure 2 were performed using a crude preparation of LPS that can signal *via* both TLR4 and TLR2. In Figure S3 in Supplementary Material, we compared activation of *Gpr84* mRNA expression induced by two different preparations of LPS, one crude (cLPS) and a more pure preparation (pLPS). Both LPS preparations gave similar levels of *Gpr84* induction at 8 h to that seen with 300 ng ml^−1^ Pam3Cys, a TLP1/2 agonist. Taken together, our macrophage expression data show that high levels of *Gpr84* mRNA expression can be induced *via* signaling *via* multiple TLRs.

### *Gpr84* Expression is Increased During Chronic Low-Grade Inflammation

**Figure 3:**
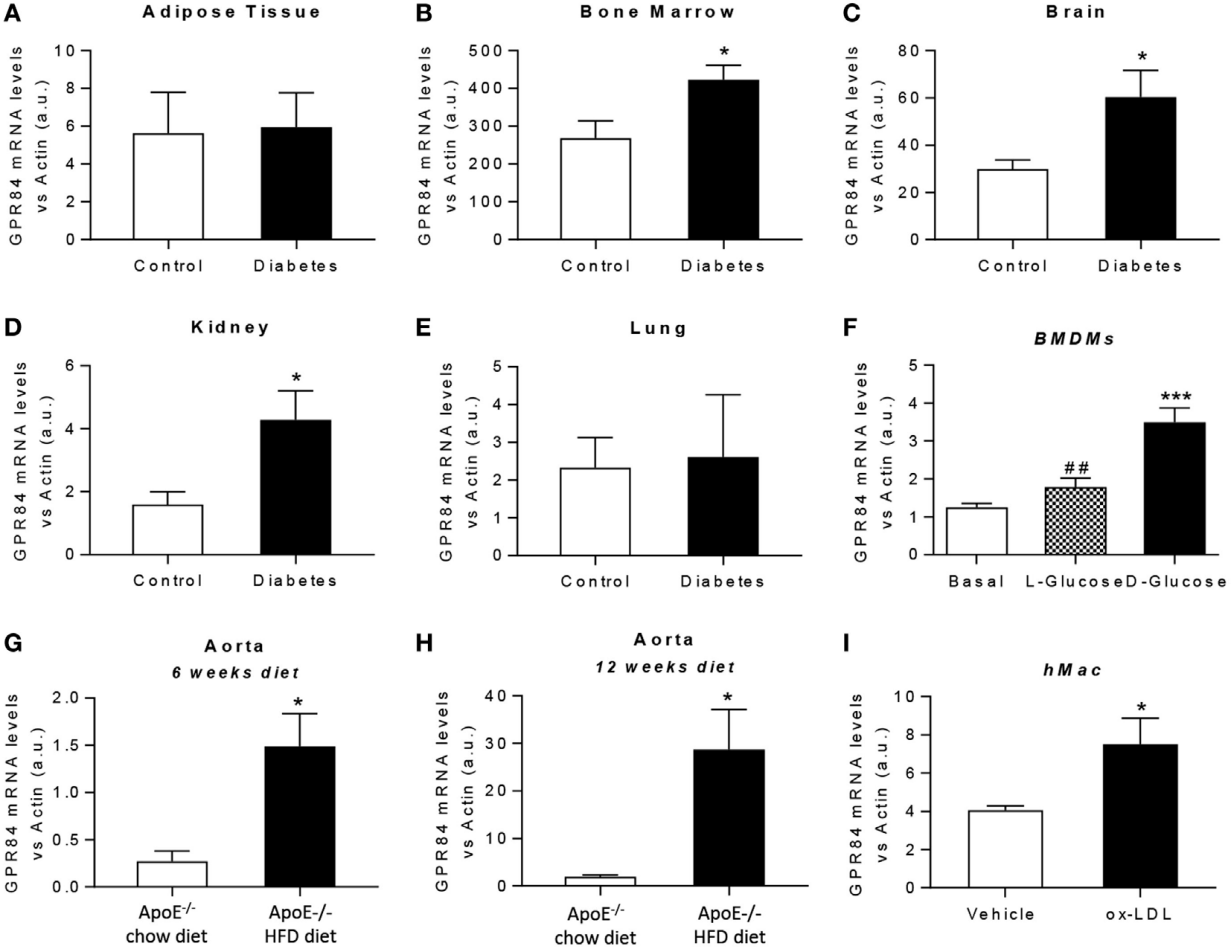
Gpr84 expression is enhanced under hyperglycemia and hypercholesterolemia conditions. **(A–E)** Non-obese diabetic mice were assessed for diabetes by blood glucose test and, once diagnosed diabetic, they were sacrificed and *Gpr84* expression was examined by q-PCR in the cDNA from adipose tissue **(A)**, bone marrow **(B)**, brain **(C)**, kidney **(D)**, and lung **(E)**. Data are presented as mean ± SEM of *n* = 4–5 mice per group. Statistical significance was assessed using a Student’s unpaired *t*-test. *P ≤ 0.05 versus control group. **(F)** mRNA expression of GPR84 receptor was analyzed by qPCR on bone marrow-derived macrophages grown in low glucose media (5 mM) following exposure to high glucose shock (30 mM) for 4 h. Error bars represent SEM of *n* = 4 separate experiments. Statistical significance was assessed using one-way ANOVA with Dunnett’s multiple comparison *post hoc* test. *P ≤ 0.05, **P ≤ 0.01, ***P ≤ 0.001 versus basal; ^#^*P* ≤ 0.05, ^##^*P* ≤ 0.01 versus d-glucose. **(G,H)** ApoE^−/−^ mice were fed either high-fat diet or chow diet. Animals were sacrificed at 6 **(G)** and 12 **(H)** weeks of diet and *Gpr84* expression was examined by q-PCR of cDNA prepared from aortic tissue. Data are presented as mean ± SEM of 4–5 mice per group. Statistical significance was assessed using a Student’s unpaired *t*-test. *P ≤ 0.05 versus ApoE^−/−^ + chow diet. **(I)** mRNA expression of GPR84 receptor was analyzed by q-PCR of human monocyte-derived macrophages challenged with oxLDL for 48 h. Error bars represent SEM of *n* = 5 separate experiments. Statistical significance was assessed using a Student’s unpaired *t*-test. *P ≤ 0.05 versus vehicle.

It is known that chronic low-grade inflammation plays a key role in the pathogenesis of atherosclerosis and diabetes (26, 27). Thus, we aimed to evaluate the expression of the GPR84 receptor under both hyperglycemic and dyslipidaemic conditions in mouse tissues and in cultured BMDMs. For *in vivo* diabetes studies, tissues were immediately harvested from NOD mice once classed as diabetic by glycosuria. Littermate age-matched NOD mice controls were those with no detectable glycosuria at the time of analysis. *Gpr84* mRNA expression was found to be significantly upregulated in the bone marrow, brain, and kidney of diabetic mice (Figures 3B–D). However, no significant changes in *Gpr84* expression were observed in adipose tissue and lung (Figures 3A,E). *In vitro*, BMDMs grown in low glucose conditions (5.5 mM) showed a significant increase in *Gpr84* expression when stimulated with high (25 mM) d-glucose, but not with l-Glucose (Figure 3F).

Atherosclerosis is a chronic inflammatory disease and to evaluate the expression of GPR84 in this condition, ApoE^−/−^ mice were fed a HFD for 6 and 12 weeks, then sacrificed and tissues collected. Male ApoE^−/−^ mice fed a chow diet were used as controls. ApoE^−/−^ HFD groups had significantly higher levels of *Gpr84* mRNA in their aortae compared to controls, at both 6 and at 12 weeks of dietary manipulation. These levels were significantly higher after 12 weeks compared to 6 weeks of HFD (Figures 3G,H). However, no differences between groups were detected in the expression of *Gpr84* in other tissues including adipose tissue, brain, kidney, and lung (data not shown). In parallel, *in vitro* studies were performed using hMDMs, which showed approximately twofold increase in *Gpr84* expression when challenged with oxidized LDL particles for 48 h (Figure 3I).

Taken together, our results suggest that the GPR84 receptor could play a role in the inflammatory process that underlies diabetes and atherosclerosis. Further studies are necessary to elucidate the specific function of the GPR84 receptor in these pathologies.

### 6-OAU Is a Specific Synthetic Ligand of GPR84 in Primary Macrophages

**Figure 4:**
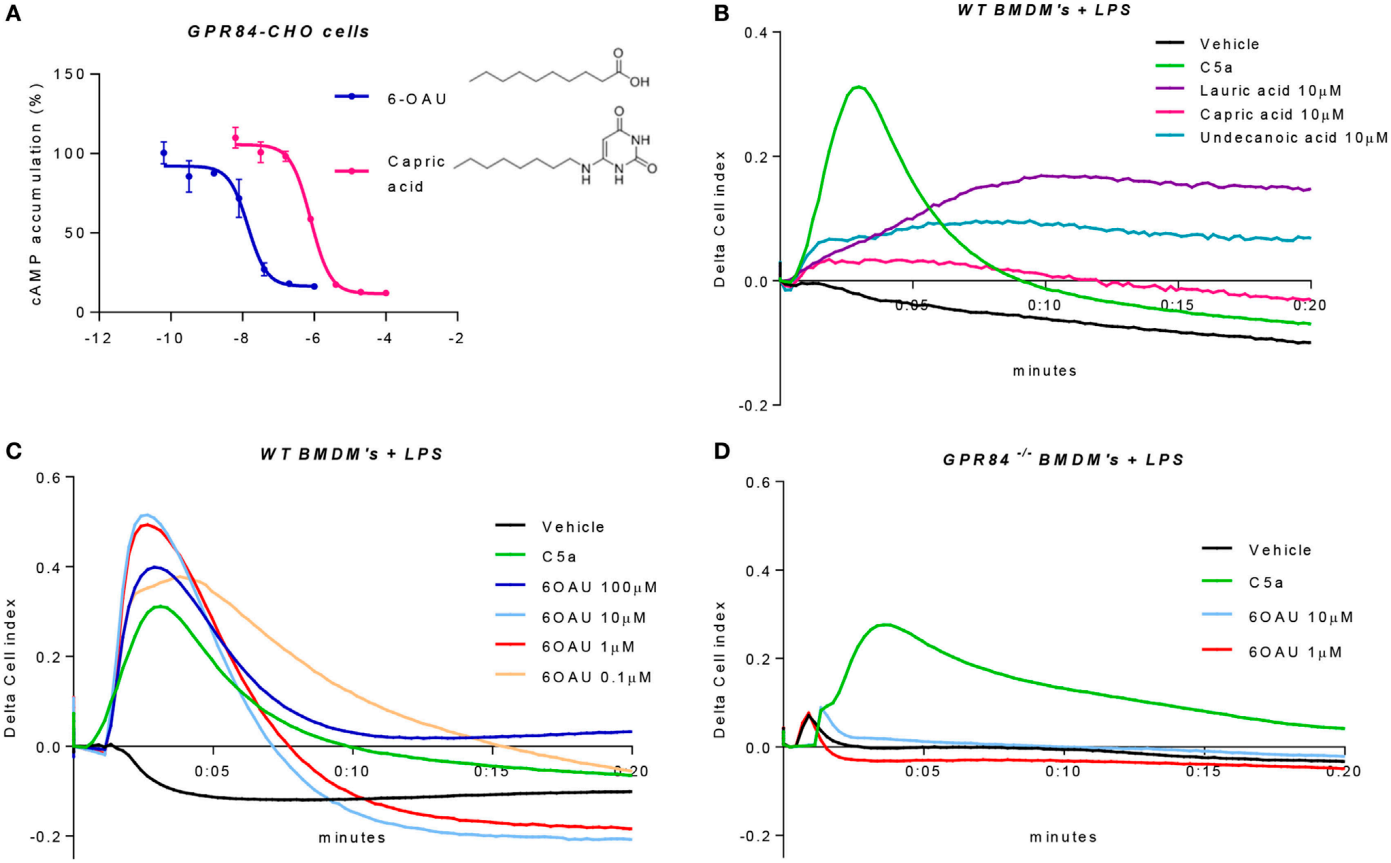
6-OAU is a potent and specific surrogate agonist of GPR84. **(A)** Intracellular cyclic AMP levels were measured in CHO-GPR84 cells following forskolin stimulation and incubation with 6-OAU or capric acid. Data are represented as the mean ± SEM of the percentage of the response to forskolin in the absence of agonists from *n* = 3 independent experiments. **(B–D)** A real-time cell impedance assay was performed to measure changes in cell impedance in response to agonists. **(B)** Bone marrow-derived macrophages (BMDMs) (50,000 cells/well) were added 0.1 µg/ml LPS and were seeded into a 96-well E-plate allowed to adhere for 16 h. Afterward, cells were treated with vehicle (0.3% DMSO), 10 nM C5a, 10 µM capric acid, 10 µM lauric acid, or 10 µM undecanoic acid. CI measurements were taken every 10 s after compound addition. LPS (0.1 µg/ml) was added to WT BMDMs **(C)** and GPR84^−/−^ BMDMs **(D)** seeded in a 96-well E-plate for 16 h before agonist addition. Cells were stimulated with either vehicle (0.3% DMSO), 10 nM C5a, or 6-OAU at the doses indicated in the legend. CI measurements were taken every 10 s after compound addition. Curves represent changes in cell index after agonist addition. Representative figures from *n* = 4 independent biological experiments are shown.

Because of the lack of consensus about whether MCFAs are the true physiological ligands of GPR84, several studies have south to develop new small molecule agonist compounds. One of these, 6-OAU, has been shown to activate GPR84 with good potency and specificity compared to MCFAs and other surrogate agonists such as diindolylmethane (DIM) (13). We, therefore, performed a cAMP assay in CHO cells transfected with the human GPR84 receptor (hereafter referred to as GPR84-CHO) to compare the potency of 6-OAU and capric acid on the activation of GPR84. When activated, GPR84 binds to the G_αi_ protein, thus inhibiting the adenylate cyclase enzyme and in turn abrogating the production of intracellular cAMP. As reported in Figure 4A, 6-OAU showed an EC_50_ = 14 nM inhibiting the production of cAMP and, being 57 times more potent than capric acid. 6-OAU showed no inhibition of cAMP in non-transfected CHO cells (data not shown).

In order to corroborate these findings in primary macrophages, we employed an electrical impedance assay that allows measurement of cytoskeletal dynamics in real-time following GPCR activation (20). For these experiments and all the signaling assays hereafter, BMDMs were pre-treated with LPS in order to place cells in an inflammatory environment and promote an increased expression of GPR84. We first assessed the effect of the three leading MCFAs that have been claimed as physiological ligands of GPR84, namely lauric acid, capric acid, and undecanoic acid. BMDMs that had been pre-treated with LPS were challenged with lauric, capric, and undecanoic acids and changes in the electrical impedance after agonist addition were detected and represented as Cell Index response curves. As shown in Figure 4B, none of the three MCFAs caused a similar BMDM response curve to that seen with C5a, a potent macrophage stimulus used as positive control and acting through a different receptor. Indeed, the macrophage response to capric acid was comparable to vehicle. We then tested the synthetic GPR84 agonist 6-OAU in BMDM signaling. Different concentrations of 6-OAU were added to LPS pre-treated BMDMs, and as shown in Figure 4C, stimulation of WT BMDMs with 6-OAU caused a rapid increase in cell index, which correlates with increased cell spreading. The maximal response of BMDMs took place when adding 1–10 µM 6-OAU and was, even higher than the response of BMDMs to C5a. In contrast, no response was observed when adding any dose of 6-OAU to GPR84^−/−^ cells, where signals were at similar levels to the vehicle (Figure 4D). However, GPR84^−/−^ cell responses to C5a were the same as WT macrophages. Thus, our data indicate that 6-OAU is a specific agonist of GPR84 and, as such, induces a biological response in primary macrophages.

### Activation of GPR84 Potentiates AKT and ERK Phosphorylation and p65 Nuclear Translocation in WT, but Not in GPR84^−/−^ BMDMs

**Figure 5:**
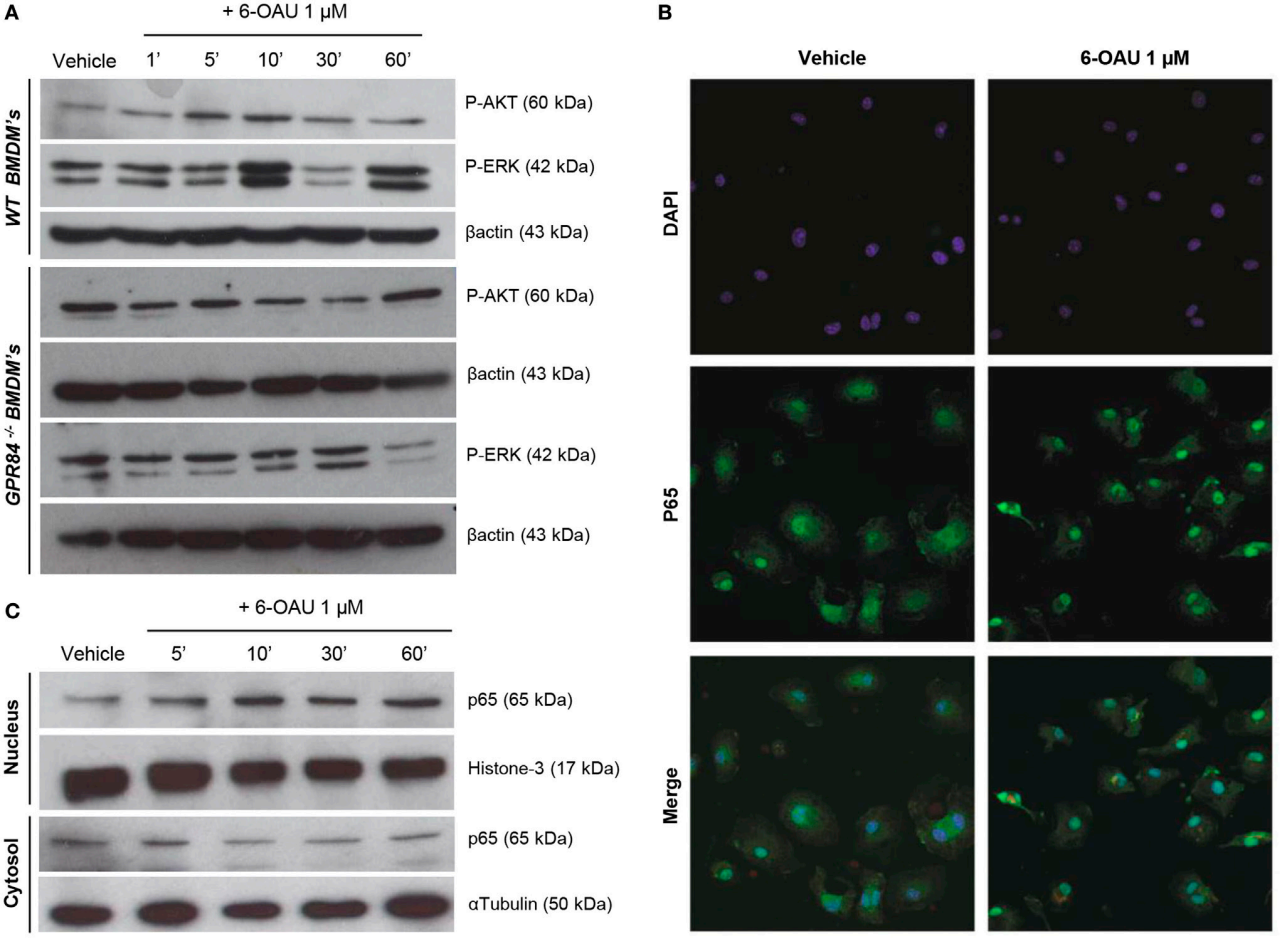
GPR84 signaling activates AKT, ERK, and NFκβ in WT, but not in GPR84^−/−^ macrophages. **(A)** Bone marrow-derived macrophages (BMDMs) were treated with 0.1 µg/ml LPS for 2 h before stimulated with either vehicle (0.3% DMSO) or 1 µM 6-OAU for 1, 5, 10, 30, and 60 min. Cell lysates were prepared and western blotting conducted for either phosphorylated Akt (P-AKT) or ERK 1/2 (P-ERK), followed by stripping and re-staining for β-actin as a loading control. Representative images from *n* = 3 independent experiments are shown. **(B)** BMDMs were treated with LPS (0.1 µg/ml) for 2 h before stimulation with vehicle (0.3% DMSO) or 1 µM 6-OAU for 30 min followed by p65 staining. Confocal microscopy images are illustrative of two separate experiments. **(C)** BMDMs were treated with LPS (0.1 µg/ml) for 2 h before stimulation with either vehicle (0.3% DMSO) or 1 µM 6-OAU for 5, 10, 30, and 60 min. Western blotting for p65 was performed using samples from cytoplasmic and nuclear fractions of cell lysates, followed by stripping and re-staining for histone-3 in the nuclear fraction and α-tubulin in the cytoplasmic fraction as a loading control. Representative images from *n* = 3 independent experiments are shown.

To investigate the potential functional significance of GPR84 activation in macrophages during the inflammatory response, we focused our attention on the main intracellular signaling cascades involved in key macrophage processes including migration, adhesion, and phagocytosis. We, therefore, analyzed the activation of AKT, ERK, and the NFκB subunit p65 in BMDMs pre-treated with 0.1 µg/ml LPS before adding 1 µM 6-OAU for different periods. As shown in Figure 5A, an increase in phosphorylation of AKT was observed when compared to vehicle in WT BMDMs just 5–10 min after 6-OAU addition. This increased activation returned to basal levels after 30 min of treatment with the agonist. Similarly, a peak of ERK phosphorylation was seen after 10 min of 6-OAU incubation. However, ERK revealed a different activation kinetic, as another peak of phosphorylation was observed at 60 min of agonist exposure. In contrast, when the same experiment was performed in GPR84^−/−^ BMDMs, no significant differences in the activation of AKT and ERK were seen when cells were treated with 6-OAU. For assessing the differential expression of the NFκB subunit p65 in BMDMs, the cellular compartments were separately analyzed by immunofluorescence and Western blot. When activated by phosphorylation, NFκB subunits translocate to the nucleus and activate the transcription of inflammatory genes (28). As shown in Figure 5B, the distribution of p65 was more concentrated to the nucleus when LPS-primed cells were treated with 6-OAU compared to vehicle, indicating NFκB activation. Western blot data confirmed that 10 min of 6-OAU treatment was the time point with maximal levels of p65 nuclear translocation (Figure 5C).

### GPR84 Activation Enhances Pro-Inflammatory Cytokine and Chemokine Expression in Macrophages

**Figure 6:**
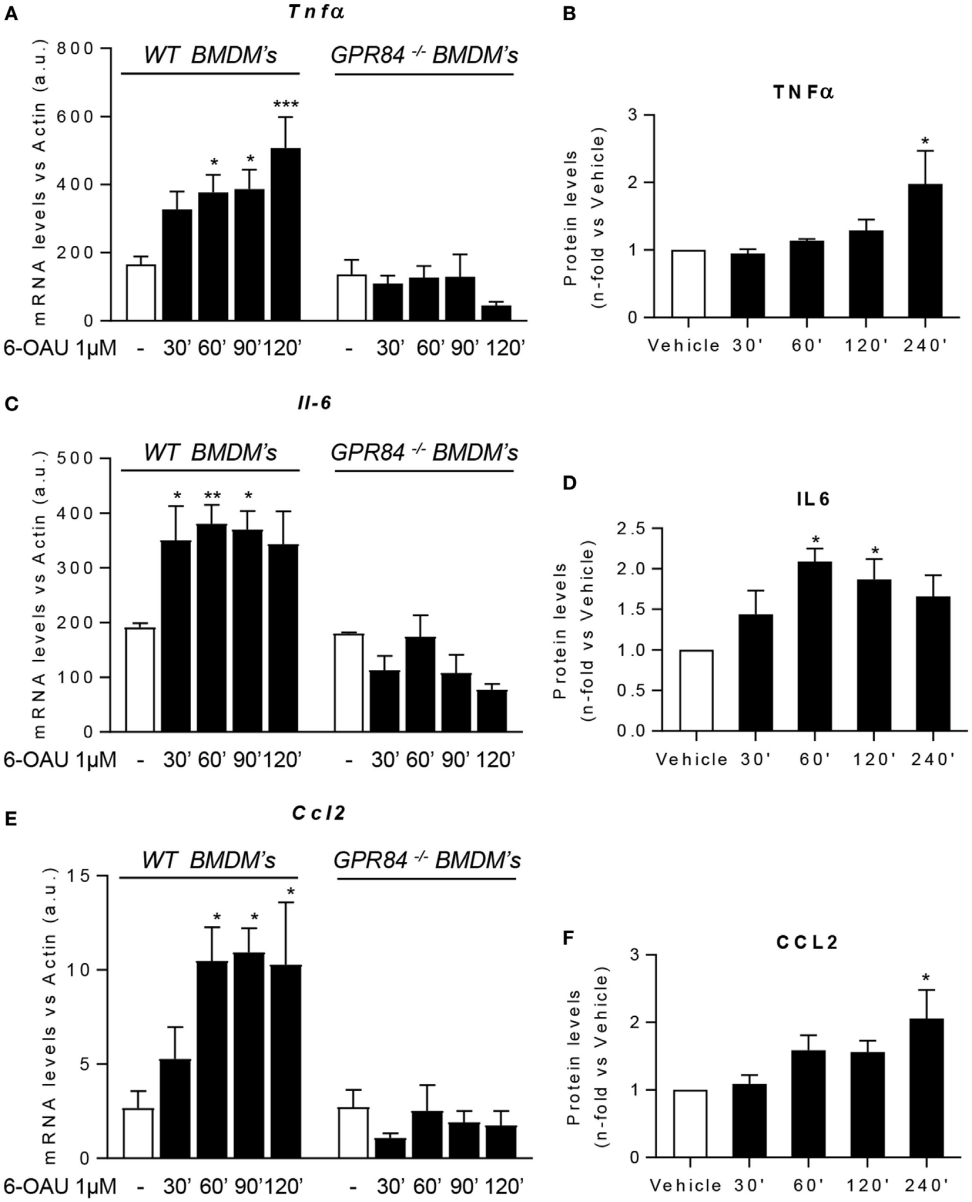
GPR84 activation enhances pro-inflammatory mediator expression in macrophages. **(A,C,E)** Bone marrow-derived macrophages (BMDMs) were treated with 0.1 µg/ml LPS for 2 h before stimulation with vehicle (0.3% DMSO) or 1 µM 6-OAU for 30, 60, 90, 120, and 240 min. mRNA expression of *Tnf*α **(A)**, *Il-6* **(C)**, and *Ccl2* **(E)** was analyzed by q-PCR of WT and GPR84^−/−^ BMDMs. Secreted protein levels of TNFα **(B)**, IL-6 **(D)**, and CCL2 **(F)** were measured by ELISA. Data presented as mean ± SEM of *n* = 4–6 separate experiments. Statistical significance was assessed using one-way ANOVA with Dunnett’s multiple comparison *post hoc* test. *P ≤ 0.05, **P ≤ 0.01, ***P ≤ 0.001 versus vehicle.

One of the major macrophage functions in inflammation is to modulate the immune response by production of cytokines, chemokines, and other inflammatory mediators (29). Since increased levels of signaling pathway activation were found in BMDMs following 6-OAU treatment, we next tested whether there was increased expression of inflammatory mediators after GPR84 activation in macrophages. We evaluated the expression of the cytokines TNFα, IL-6, and IL-12b. We also analyzed the expression of the chemokines CCL2, CCL5, and CXCL1, the scavenger receptor FCγRI, and the adhesion molecule ICAM-1 (30). Measurement of these mediators by qRT-PCR showed that 6-OAU-mediated-GPR84 activation promoted enhanced expression of inflammatory cytokines and chemokines in LPS pre-treated WT BMDMs compared with vehicle-treated cells. *Tnf*α levels progressively increased with time (Figure 6A), while *Il-6* (Figure 6C), *Ccl2* (Figure 6E), *Il-12b, Ccl5, Cxcl1, Fc*γ*RI*, and *Icam-1* (Figures S4A–E in Supplementary Material) levels rapidly increased at 30–60 min post 6-OAU addition and remained elevated at later time points. However, as shown in Figure S5 in Supplementary Material, naïve WT BMDMs—without LPS pre-treatment—did not show an upregulation of inflammatory gene expression mediated by 6-OAU alone, most likely explained by the low expression levels of the GPR84 receptor. Importantly, GPR84^−/−^ BMDMs did not show any upregulated gene expression when treated with 6-OAU at any time point, thus indicating that all the effects found in WT cells stimulated with 6-OAU were GPR84-mediated (Figures 6A,C,E).

In addition, not only gene expression but also protein levels measured by ELISA were enhanced post GPR84 activation (Figures 6B,D,F). Whereas TNFα and CCL2 protein secretion peaked after 4 h post 6-OAU addition, IL-6 showed highest levels at just 1 h. Taken together, our data show that pro-inflammatory mediator production in LPS-primed macrophages is rapidly upregulated upon GPR84 activation by a synthetic agonist.

### GPR84 Blockade With a Selective Antagonist Counteracts the Enhanced Inflammatory Effects Mediated by 6-OAU in Macrophages

**Figure 7:**
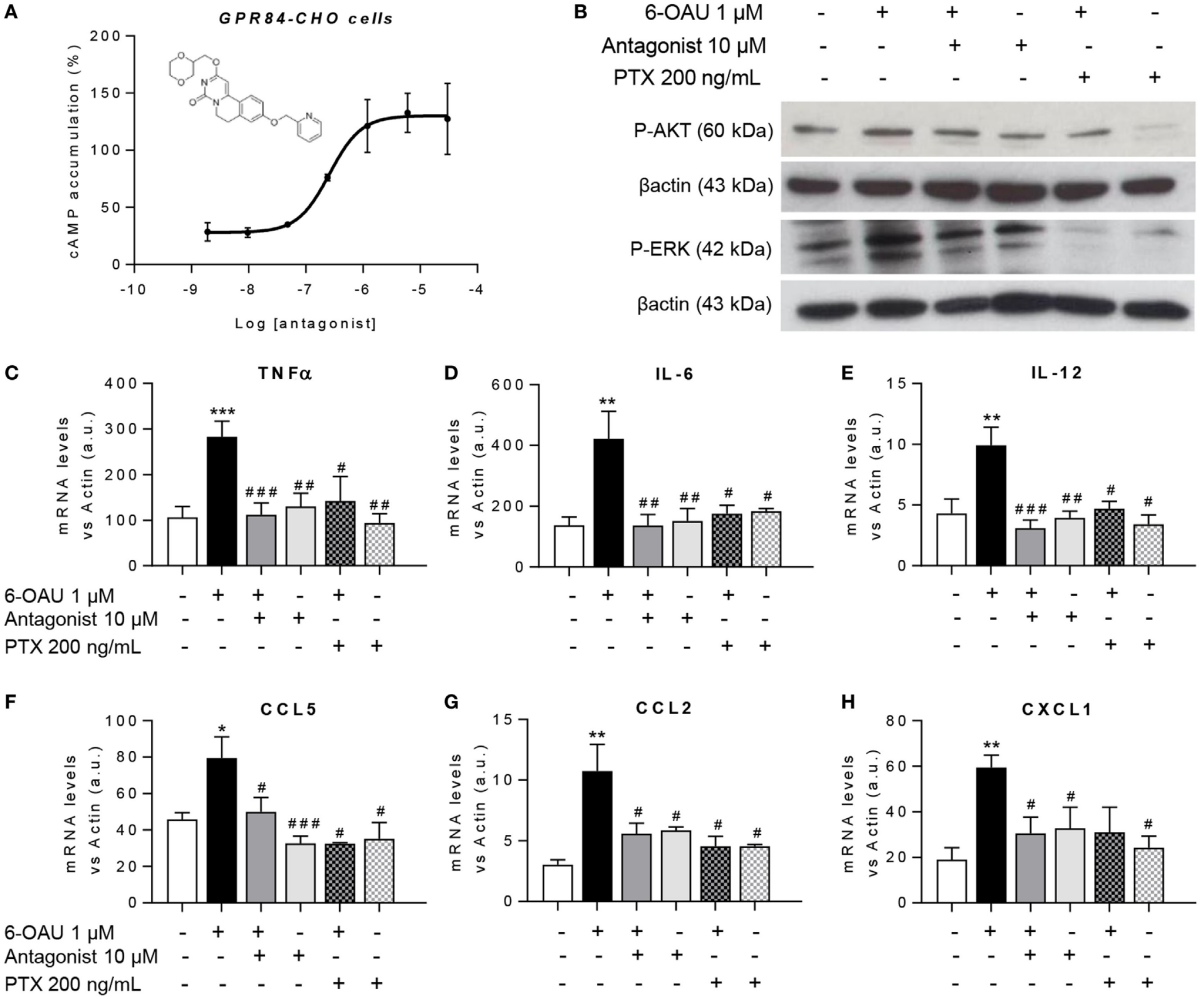
GPR84 antagonist abrogates the enhanced inflammatory response mediated by 6-OAU. **(A)** Intracellular cyclic AMP levels were measured in CHO-GPR84 cells pre-treated with the antagonist followed by forskolin and 6-OAU stimulation. Data are represented as the mean ± SEM of the percentage of the response to forskolin in the absence of agonists *n* = 3 independent experiments. **(B–H)** Bone marrow-derived macrophages were treated with LPS (0.1 µg/ml) for 2 h before pre-treatment with vehicle (0.3% DMSO), 10 µM antagonist for 30 min, or 200 ng/ml Pertussis toxin for 90 min. Afterward cells were stimulated with either vehicle (0.3% DMSO) or 1 µM 6-OAU for either 10′ for protein isolation or 1 h for RNA extraction. **(B)** Cell lysates were prepared and western blotting conducted for either phosphorylated Akt (P-AKT) or ERK 1/2 (P-ERK), followed by stripping and re-staining for β-actin as a loading control. Representative images from *n* = 3 independent experiments. **(C–H)** mRNA expression of *Tnf*α **(C)**, *Il-6* **(D)**, *Il-12* **(E)**, *Ccl5* **(F)**, *Ccl2* **(G)**, and *Cxcl1* **(H)** was analyzed by q-PCR. Data are presented as mean ± SEM of *n* = 4–7 separate experiments. Statistical significance was assessed using one-way ANOVA with Dunnett’s multiple comparison *post hoc* test. *P ≤ 0.05, **P ≤ 0.01, ***P ≤ 0.001 versus vehicle; ^#^*P* ≤ 0.05, ^##^*P* ≤ 0.01, ^###^*P* ≤ 0.001 versus 6-OAU.

To test the hypothesis that blocking the activation of GPR84 could be a potential anti-inflammatory strategy in different inflammatory diseases, we used a reported GPR84 antagonist from the patent literature (Galapagos NV Company), the chemical structure of which is shown in Figure 7A. We first assessed the potency and specificity of the antagonist using GPR84-CHO cells in the cAMP assay. Our data show that the antagonist effectively inhibited the action of 6-OAU in decreasing cAMP production in GPR84-CHO cells (Figure 7A). To test this antagonist compound’s inhibition of the pro-inflammatory effects of GPR84 activation in macrophages, LPS pre-treated BMDMs were incubated with 10 µM antagonist for 30 min before adding 1 µM 6-OAU. Protein analysis by Western Blot showed that the GPR84 antagonist partially blocked the phosphorylation of AKT and ERK induced by 6-OAU (Figure 7B). Likewise, GPR84 blockade reduced the expression of TNFα, IL-6, IL12b, CCL2, CCL5, and CXCL1 in BMDMs (Figures 7C–H). PTX was used as a positive control for the inhibition of GPR84 signaling.

### 6-OAU Mediated—GPR84 Activation Promotes Migration, Bacterial Adhesion, and Phagocytosis in Macrophages, Effects That Are Inhibited by a GPR84 Antagonist

Having shown that LPS-primed BMDMs treated with a GPR84-specific agonist displayed an exacerbated inflammatory response, we next sought to investigate if these changes in protein and gene expression are associated with different functional behaviors in macrophages. During the inflammatory response, macrophages migrate to sites of inflammation and, adhere to particulate debris, which is removed by phagocytosis (31). We, therefore, performed different assays in order to evaluate changes in macrophage functionality following GPR84 activation with its agonist 6-OAU.

**Figure 8:**
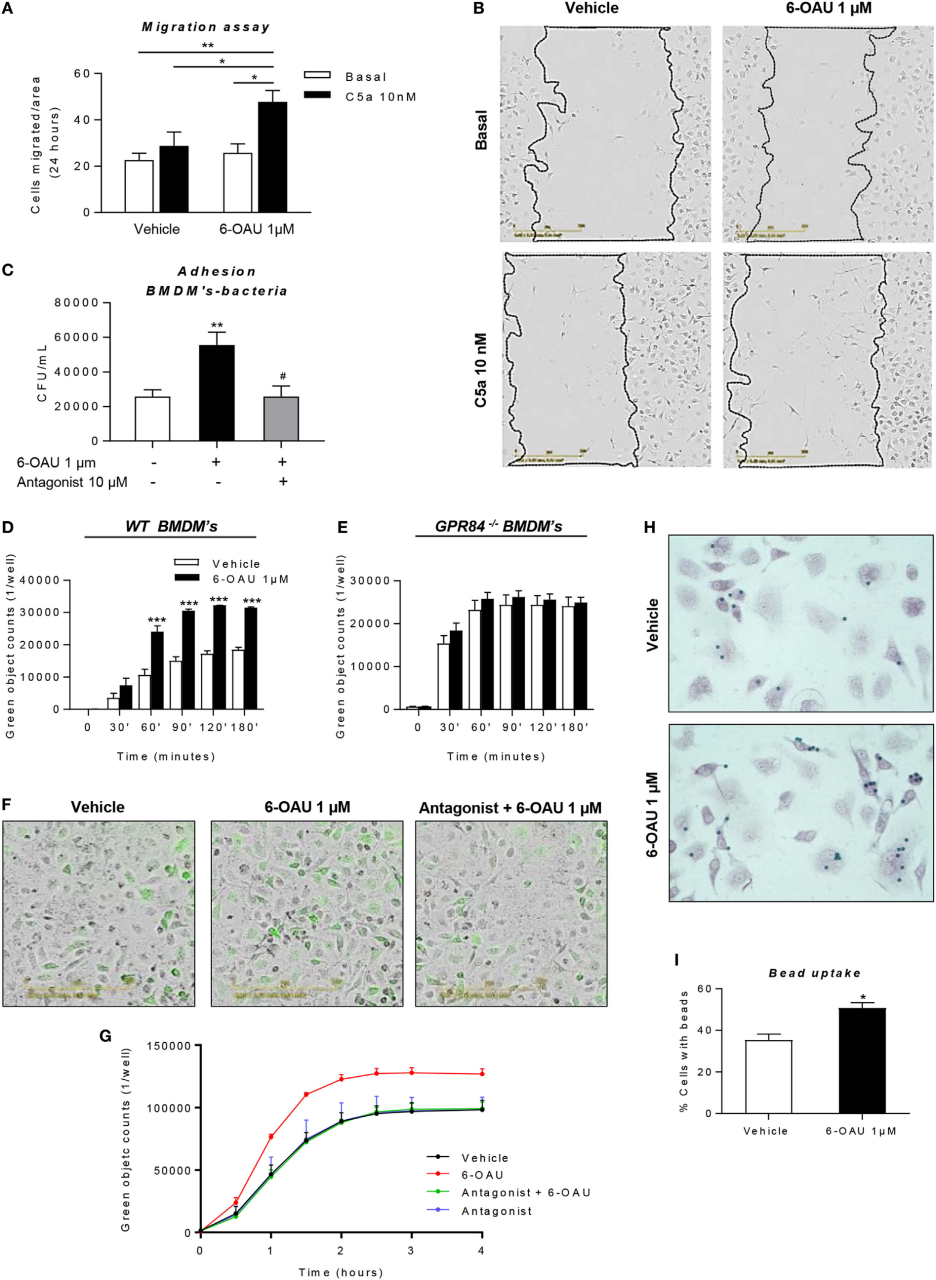
6-OAU mediated—GPR84 activation promotes macrophage migration, bacterial adhesion, and phagocytosis. Bone marrow-derived macrophages (BMDMs) were treated with LPS (0.1 µg/ml) for 2 h before stimulation with vehicle (0.3% DMSO) or 1 µM 6-OAU for 1 h. In inhibition experiments, cells were pre-treated with 10 µM antagonist before stimulation. **(A,B)** A near confluent monolayer of BMDMs was scratched and 10 nM C5a or vehicle was added to cells. **(A)** Quantification of the number of cells that had migrated into the scratch at *t* = 24 h. Data are presented as mean ± SEM of *n* = 4 separate experiments. Statistical significance was assessed using two-way ANOVA with Tukey’s multiple comparison test. **(B)** Images from the IncuCyte imaging software at 20× magnification showing cells invading the scratch after 24 h monitoring. Representative images from *n* = 4 independent experiments are shown. **(C)** *E. coli* strain DH5-α bacteria adherence to the macrophage surface were measured following the protocol given in the Section “Materials and Methods” and expressed as CFU per milliliter after BMDM lysis. **(D–G)** BMDMs were incubated with pH-rodo Green *E. coli* bioparticles (0.1 mg/ml), and fluorescence emission was measured in the IncuCyte imaging platform every 15 min for 4 h at 37°C. **(D,E,G)** Quantification of the green object counts per well (p96-well plate) in WT **(D)** and GPR84^−/−^ **(E)** BMDMs. **(G)** Green object counts per well (p24-well plate) in WT BMDMs under inhibitory conditions. Data are presented as mean ± SEM from *n* = 4 biological replicates in technical duplicate. **(F)** Representative images from the IncuCyte software from one of *n* = 4 separate experiments are shown. Scale bar 200 µm. **(H,I)** BMDMs were incubated with opsonized 3 µm polystyrene beads for 1 h at 37°C and then fixed and stained with safranin. Twenty images per well were taken and bead numbers were quantified by an observer blind to treatment group. Data are presented as the number of cells with beads inside divided by the total number of cells per image. *P ≤ 0.05, **P ≤ 0.01, ***P ≤ 0.001 versus vehicle.

As shown in Figures 8A,B, we carried out a scratch assay on BMDM monolayers, which revealed that 6-OAU-mediated GPR84 activation potentiated BMDM migration stimulated by C5a. However, this only happened in the presence of C5a, thus indicating that GPR84 signaling enhances but does not mediate macrophage migration directly. We, therefore, evaluated 6-OAU as a macrophage chemoattractant in a well-validated real-time chemotaxis assay. 6-OAU displayed no chemotactic properties when compared to C5a at any of the tested doses in BMDMs (Figure S6 in Supplementary Material).

In a live bacteria phagocytosis assay, it was found that macrophages pre-treated with 6-OAU showed an increased adhesion of GFP-labeled *E. coli* to the cell surface (Figure 8C). In contrast, cells pre-treated with the GPR84 antagonist before exposure to 6-OAU showed a similar number of adherent bacteria to vehicle treated cells.

To further assess the phagocytic activity of macrophages, we performed a pHrodo-conjugated *E. coli* phagocytosis assay using the IncuCyte ZOOM real-time cell imaging system as described previously (32). By taking images of the green fluorescence emitted by bacteria once inside the phagolysosome, we found that 6-OAU significantly enhanced phagocytosis in WT BMDMs, but not in GPR84^−/−^ BMDMs (Figures 8D,E). Furthermore, as shown in Figures 8F,G, these effects were abolished when cells were pre-treated with the GPR84 antagonist.

In a third macrophage phagocytosis assay, we examined macrophage uptake of opsonized polystyrene beads. In this bead phagocytosis assay, we found that 6-OAU mediated-GPR84 activation enhanced the number of cells phagocytosing beads, when compared to vehicle-treated cells (Figures 8H,I). However, no significant differences were observed in the number of beads per cell within those cells that had phagocytosed beads (data not shown).

## Discussion

GPR84 is a metabolic GPCR the expression of which has been previously described in leukocytes (11). However, potential roles for GPR84 signaling in modulating the inflammatory response have not been fully explored. In the present study, we show that the GPR84 receptor is upregulated in murine macrophages under inflammatory conditions *in vitro*, in hMDMs *ex vivo*, and in mouse models of endotoxemia and immuno-metabolic stress *in vivo*. We show that the synthetic GPR84 agonist 6-OAU selectively activates the GPR84 receptor in WT, but not in GPR84^−/−^ macrophages, displaying increased potency as a GPR84 agonist than the claimed physiological ligands MCFAs. Activation of GPR84 by 6-OAU enhances the inflammatory response in macrophages by increasing the phosphorylation of Akt and, ERK as well as the nuclear translocation of NFκB, causing an increase in expression of key inflammatory cytokines and chemokines. Our experiments show that 6-OAU-mediated-GPR84 activation promotes macrophage migration, bacterial adhesion, and phagocytic activity of BMDMs, but not chemotaxis. Finally, specific antagonism of GPR84 attenuates the enhanced inflammatory responses mediated by 6-OAU.

It has been previously reported that leukocyte GPR84 receptor expression is upregulated after LPS stimulation *in vitro* (11, 13). We report that in a murine model of low-dose endotoxemia, all tissues assessed displayed a significantly increased expression of *Gpr84* at 2 and 8 h post LPS injection. Bone marrow was the tissue showing the highest levels of receptor expression, which is in accordance with the GPR84 receptor being upregulated in immune cells in the context of acute inflammation (13). Specifically, we have shown that within the bone marrow, monocytes are the cell type accounting for the large increase in *Gpr84* expression after LPS treatment. We showed that not only BMDMs but also many other macrophage populations including biogel-elicited macrophages, resident peritoneal macrophages, the murine macrophage cell line RAW 264.7, mouse microglia, and human MDM, expressed higher levels of *Gpr84* when stimulated with LPS. However, we showed that this response is not unique and specific of TLR4 receptor, since other stimuli including zymosan (TLR2 and Dectin-1 specific agonist) and Pam3CysK (TLR2 specific agonist) also significantly activated *Gpr84* expression. In contrast, all macrophage populations tested showed lower expression of the receptor after IL-4 treatment. This expression pattern suggests a possible role for GPR84 in M1 polarized macrophages. However, when we evaluated the expression of BMDM *Gpr84* mRNA after LPS addition with the cytokine IFNγ (the classic M1 polarizing stimulus), we saw a consistently lower level of Gpr84 mRNA expression. It is unfortunate that we cannot study GPR84 protein expression in macrophages directly due to the lack of specificity of all commercially available anti-GPR84 antibodies tested (Figure S1 in Supplementary Material). In their review of the M1 M2 macrophage differentiation paradigm, Martinez and Gordon explicitly define macrophages treated with endotoxin as a class of macrophages distinct from “classic” M1 macrophages induced by treatment with LPS and IFNγ (33). Monoclonal antibodies that specifically recognize GPR84 could be a good marker for a population of activated macrophages distinct from M1 macrophages at sites of inflammation *in vivo*.

Considering that GPR84 has been claimed as a metabolic GPCR, we wanted to evaluate how its expression was regulated in two important metabolic diseases where inflammation plays a key role: diabetes and atherosclerosis (Figure 3). The pattern of *Gpr84* expression in tissues of NOD mice that had recently become diabetic is consistent with pathological changes in hematopoiesis (bone marrow) and the kidney. Increased brain expression *Gpr84* mRNA is interesting but difficult to interpret without looking at RNA and protein expression in mouse disease models and human tissue. However, this correlates with the previously reported high *Gpr84* expression in microglia in different disease models such as endotoxemia, experimental autoimmune encephalomyelitis, Alzheimer, and neuronal injury (34–36). *Gpr84* mRNA levels where elevated in aortic tissue of ApoE^−/−^ mice fed a high fat diet rather than a regular chow diet (Figures 3G,H) strongly suggesting elevated *Gpr84* expression in atherosclerotic lesions. We speculate that the source of the increased *Gpr84* content could be from infiltrating macrophages into the lesion. Furthermore, our data showing that macrophages stimulated with oxidized LDL particles expressed high levels of *Gpr84* suggest that infiltrating macrophages could be involved in the atherosclerotic plaque progression (26). Our demonstration that synthetic agonists of GPR84 activate pro-inflammatory signaling and cytokine release in already activated macrophage populations suggests that detailed examination of GPR84 expression and signaling is merited in murine and human atherosclerosis. Given the lack of macrophage signaling in response to capric acid seen in our cell impedance assays (Figure 4B), atherosclerotic lesions may be a good place to start looking for physiological ligands active at this enigmatic receptor.

In the continuing debate over the true physiological agonist(s) for the GPR84 receptor, researchers are placing increased reliance on chemical tool compounds to explore the role of GPR84 signaling in leukocyte biology (37). In this study, we chose to use the synthetic agonist 6-OAU rather than two other GPR84 synthetic agonists embelin (38, 39) and DIM (12, 15). This was because 6-OAU is so far, the most specific GPR84 agonist. Embelin has been reported to potently inhibit the X-linked inhibitor of apoptosis protein in cancer cells (40–42) and has been shown to bind the Adenosine A3 receptor and CXCR2 (38). Diindolylmethane 2 (DIM) is an agonist at the cannabinoid receptor 2 (CB2) in macrophages (43).

When exploring the biological effects of GPR84 agonists in macrophage biology, it is important to ensure that any effects seen with tool compounds are specific to the GPR84 receptor. We demonstrated the specificity of 6-OAU: biologically, using macrophages from GPR84^−/−^ mice, and chemically using the reported Galapagos GPR84 antagonist. We show for the first time that 6-OAU signaling is completely ablated in GPR84^−/−^ BMDMs in a number of different signaling assays including cell impedance sensing (Figure 4D), AKT, and ERK phosphorylation (Figure 5A) NFκB activation (Figures 5B,C), cytokine and chemokine expression (Figure 6), and macrophage phagocytosis (Figure 8E). These experiments cannot exclude the possibility that 6-OAU signals at other GPCRs expressed in macrophages, but they clearly demonstrate that all of 6-OAU’s biological effects on macrophages are lost when the GPR84 receptor is deleted. A similar demonstration of the GPR84 specificity of embelin and DIM is currently missing. We have shown that the GPR84 antagonist blocks the biological effects of 6-OAU in transfected GPR84-CHO cells (Figure 7A). We have further demonstrated the antagonist’s potential utility to target GPR84 in AKT and ERK phosphorylation (Figure 7B) cytokine and chemokine expression (Figures 7C–H), bacterial adhesion (Figure 8C), and macrophage phagocytosis (Figures 8F,G).

Activation of the GPR84 receptor in microglia by embelin has been shown to induce cytoskeletal changes that was interpreted as “membrane ruffling” despite an absence of live cell imaging (39). We report for the first time the ability of 6-OAU to enhance macrophage phagocytosis of pHrodo labeled *E. coli* bioparticles, an effect that is lost in GPR84^−/−^ macrophages and macrophages treated with a GPR84 antagonist. 6-OAU treatment enhances macrophage phagocytosis of opsonized polystyrene beads and doubles *E. coli* adhesion to macrophages (Figure 8C). Analysis of BMDM gene expression following 6-OAU treatment shows significantly enhanced FcγR1 mRNA expression within 30 min, which could contribute to the observed enhanced macrophage binding to bacteria (Figure S2D in Supplementary Material).

In our study, we have focused on GPR84 expression and function in murine and human macrophage populations. Our *Gpr84* mRNA expression studies could be extended to dendritic cells, lymphocytes, and neutrophils, but such studies would be more useful if they could be combined with GPR84-specific antibody reagents or fluorescently labeled GPR84 agonist compounds. Although 6-OAU has been used *in vivo* in rats, no pharmacokinetic or pharmacodynamic data are available for this GPR84 agonist (13), so *in vivo* delivery of GPR84 agonists in mice must await a full evaluation of GPR84-specific compounds in wild-type and *Gpr84*^−/−^ mice.

Our detailed studies confirm that macrophage *Gpr84* mRNA expression is significantly upregulated by pathogen molecular patterns including LPS, Pam3CysK, and zymosan. Our functional studies using the synthetic ligand 6-OAU show that murine macrophages pre-treated with LPS exhibit enhanced inflammatory signaling following GPR84 activation resulting in increased expression of key inflammatory cytokines and chemokines. In contrast, macrophages do not respond significantly to 6-OAU in the absence of LPS pre-treatment. This can be explained by the fact that expression levels of *Gpr84* mRNA in basal conditions are relatively low compared to those seen in LPS conditions, hence limiting GPR84 agonist signaling *via* its cognate receptor and induction of downstream signaling.

In contrast to previous published work, we find no evidence that GPR84 agonists act as chemoattractants for murine macrophages. Rather, 6-OAU enhances macrophage phagocytosis of opsonized and unopsonised targets, an effect that is lost in *Gpr84*^−/−^ cells and in macrophages pre-treated with a published GPR84 antagonist.

Our novel findings are consistent with a model in which GPR84 acts as a receptor that can amplify macrophage inflammatory responses at sites of inflammation and hence could represent an interesting target for the development of novel anti-inflammatory or antiatherogenic therapeutics.

## Author Contributions

CR, DLu, GP, PI, LZ, AI, DLi, CO, LuD, EG, AR, GW, LeD, CM, and DG performed experiments; CR, PI, GP, and LZ analyzed results and made the figures; CR and DG designed the research, and wrote the paper. All authors provided critical revision of the manuscript.

## Conflict of Interest Statement

The authors declare that the research was conducted in the absence of any commercial or financial relationships that could be construed as a potential conflict of interest.

## Abbreviations

6-OAU, 6-(octylamino) pyrimidine-2,4(1H,3H)-dione/6-*n*-octylaminouracil; ApoE^−/−^, apolipoprotein E knockout; BMDMs, bone marrow-derived macrophages; cAMP, cyclic AMP; CI, Cell Index; DAG, diacylglycerol; DIM, diindolylmethane 2,; FBS, fetal bovine serum; FFAs, free fatty acids; GPCR, G protein-coupled receptor; GPR84-CHO, CHO cells transfected with the human GPR84 receptor; HFD, high-fat diet; hMDMs, human monocyte-derived macrophages; i.p., intraperitoneal; IP3, inositol triphosphate; MCFA, medium chain fatty acid; NFκB, nuclear factor κB; NOD, non-obese diabetic; PAMPs, pathogen-associated molecular patterns; PTX, pertussis toxin; PMNs, polymorphonuclear leukocytes; PBMC, peripheral blood mononuclear cells; TBS-T, tris-buffered saline with Tween.
