# Supplementary material for: Activation of the Immune-Metabolic Receptor GPR84 Enhances Inflammation and Phagocytosis in Macrophages
Source: Front Immunol. 2018 Jun 20;9:1419. doi: 10.3389/fimmu.2018.01419 (PMC6019444; doi:10.3389/fimmu.2018.01419)

Supplementary Material

Activation of the Immune-Metabolic Receptor GPR84 Enhances Inflammation and Phagocytosis in Macrophages.

Carlota Recio[1], Daniel Lucy[1,2], Gareth S.D. Purvis[1], Poppy Iveson[1], Lynda Zebouji[1], Asif J. Iqbal[1], Daniel Lin[3], Chris O'Callaghan[3], Lucy Davison[3], Esther Griesbach[1], Angela J. Russell[2,4], Graham M. Wynne[2], Lea Dib[5], Claudia Monaco[5], David R. Greaves*[1]

[1] Sir William Dunn School of Pathology, University of Oxford, Oxford, United Kingdom.
[2] Department of Chemistry, University of Oxford, Oxford, United Kingdom.
[3] Nuffield Department of Medicine, Wellcome Trust Centre for Human Genetics, University of Oxford, Oxford, United Kingdom.
[4] Department of Pharmacology, University of Oxford, Oxford, United Kingdom.
[5] Kennedy Institute of Rheumatology, University of Oxford, Oxford, United Kingdom.

***Correspondence:**

David R. Greaves, Sir William Dunn School of Pathology, University of Oxford, Oxford OX1 3RE, United Kingdom. Phone: +44 1865 285519; email: david.greaves@path.ox.ac.uk

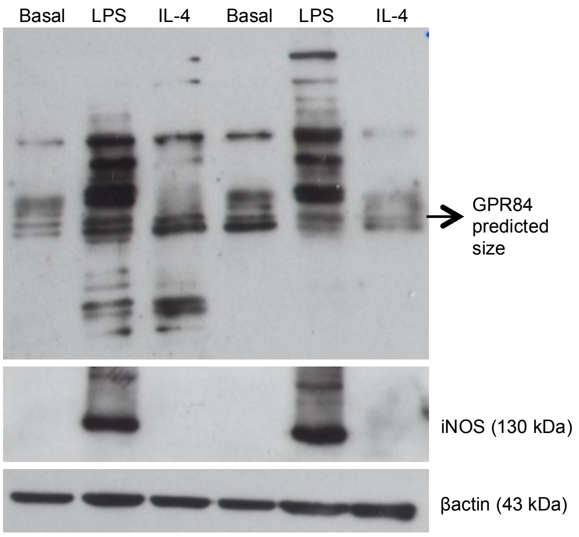

Supplementary Figure 1. Lack of specific antibodies against murine GPR84 receptor.

GPR84 protein expression was analysed by western blotting in total cell lysates of BMDMs from two independent mice challenged with either LPS or IL-4 for 16 hours. Monoclonal antibodies from multiple commercial suppliers gave similar results.

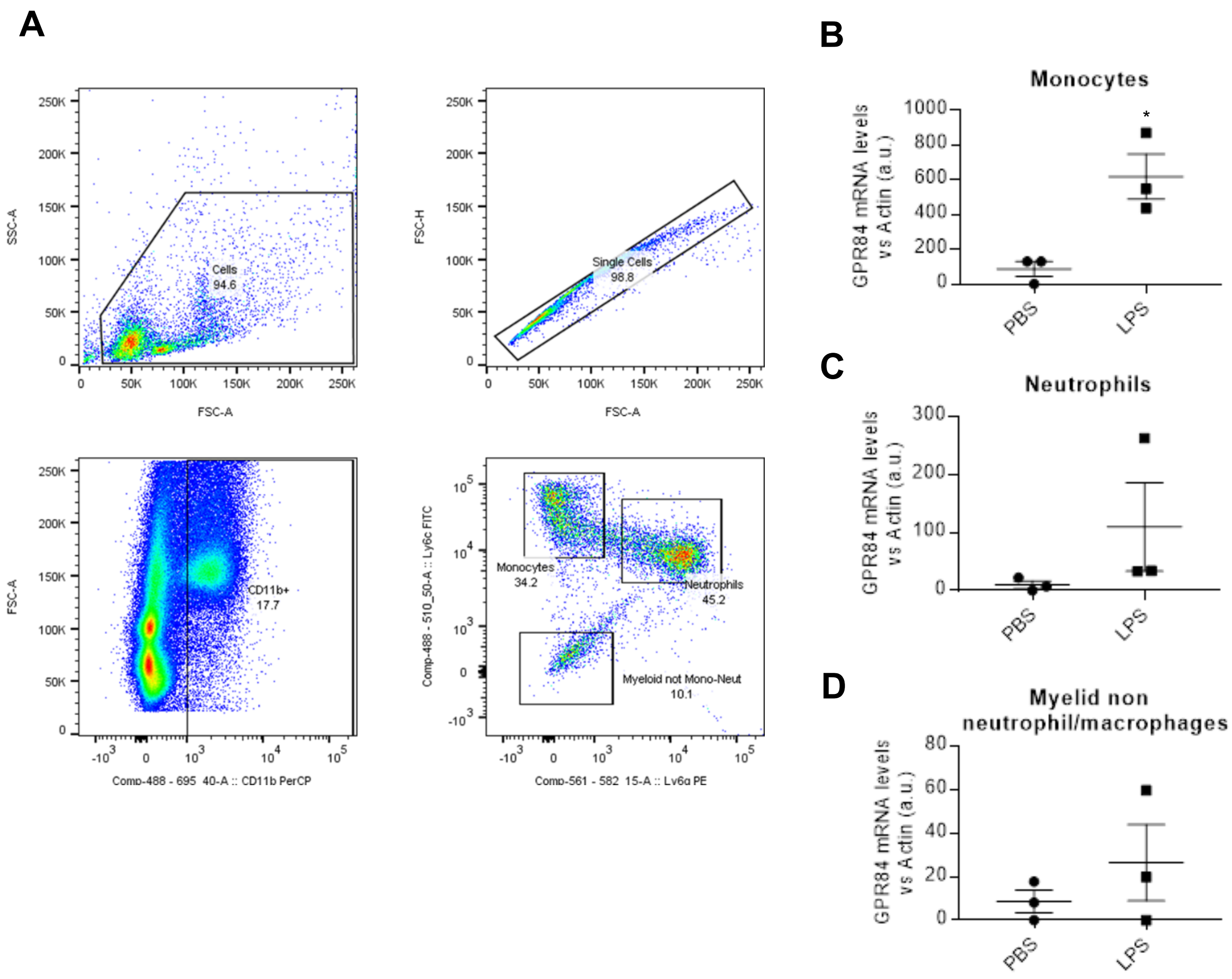

Supplementary Figure 2. Monocytes account for the increase in *Gpr84 mRNA* expression in bone marrow post LPS stimulation.

Monocytes, neutrophils and myeloid cells -not monocytes or neutrophils- from mice treated with LPS (1 mg/kg) or vehicle (PBS) for 2 hours were sorted using FACS. Representative gating for FACS of bone marrow for isolation (**A**), mRNA expression of *gpr84* in monocytes (**B**), neutrophils (**C**) and myeloid cells non neutrophil/monocytes (**D**). Statistical significance was assessed using one-way ANOVA with Dunnett´s multiple comparison posthoc test. * $P \leq 0.05$ versus vehicle.

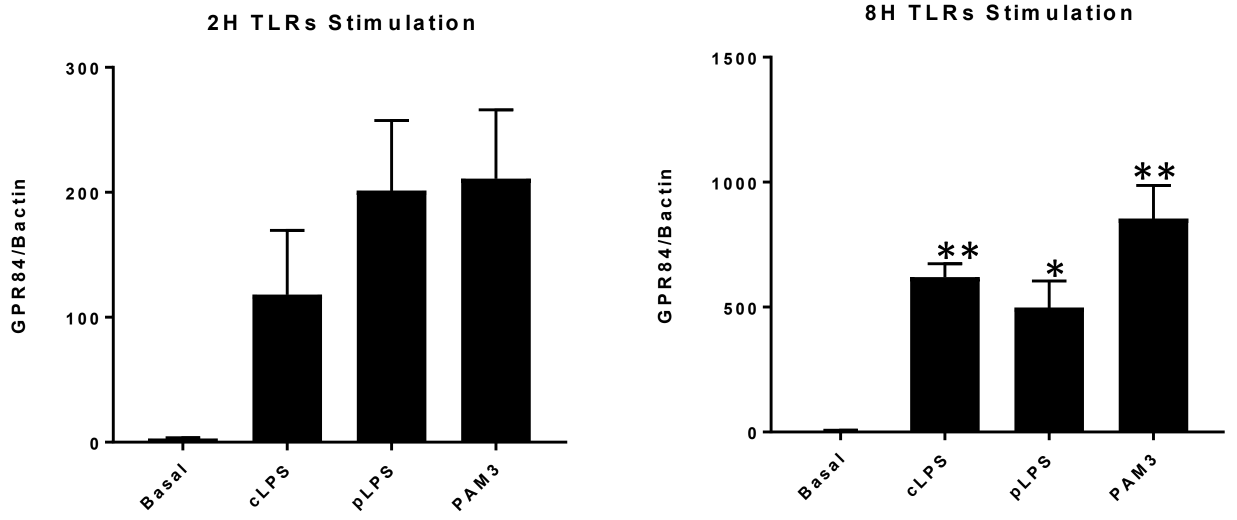

Supplementary Figure 3. Up-regulation of *Gpr84* mRNA expression is not specific to TLR4.

BMDMs were treated with crude LPS (cLPS - 0.1 µg/ml), or a more more purified preparation of LPS (pLPS - 0.1 µg/ml) and Pam3Cysk (PAM - 0.3 µg/ml) for 2 hours (**A**), or 8 hours (**B**) and mRNA expression of *Gpr84* was analysed by q-PCR. Data presented as mean ± S.E.M of n = 4 biological replicates. Statistical significance was assessed using one-way ANOVA with Dunnett´s multiple comparison posthoc test. * $P \leq 0.05$ versus vehicle.

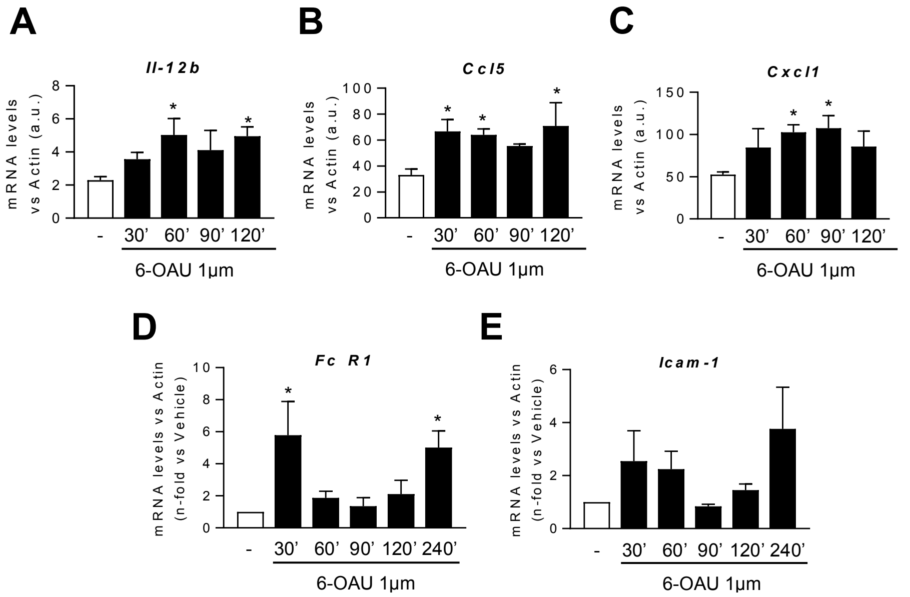

Supplementary Figure 4. GPR84 activation enhances pro-inflammatory mediator expression in macrophages.

BMDMs were treated with LPS (0.1 μg/ml) for 2 hours before stimulation with either vehicle (0.3% DMSO) or 1 μM 6-OAU for 30, 60, 90, and 120 minutes. mRNA expression of *Il-12b* (**A**), *Ccl5* (**B**), *Cxcl1* (**C**), *FcγRI* (**D**) and *Icam-1* (**E**) was analysed by q-PCR on WT BMDMs. Data presented as mean ± S.E.M of n = 4-6 separate experiments. Statistical significance was assessed using one-way ANOVA with Dunnett´s multiple comparison posthoc test. * P ≤ 0.05 versus vehicle;

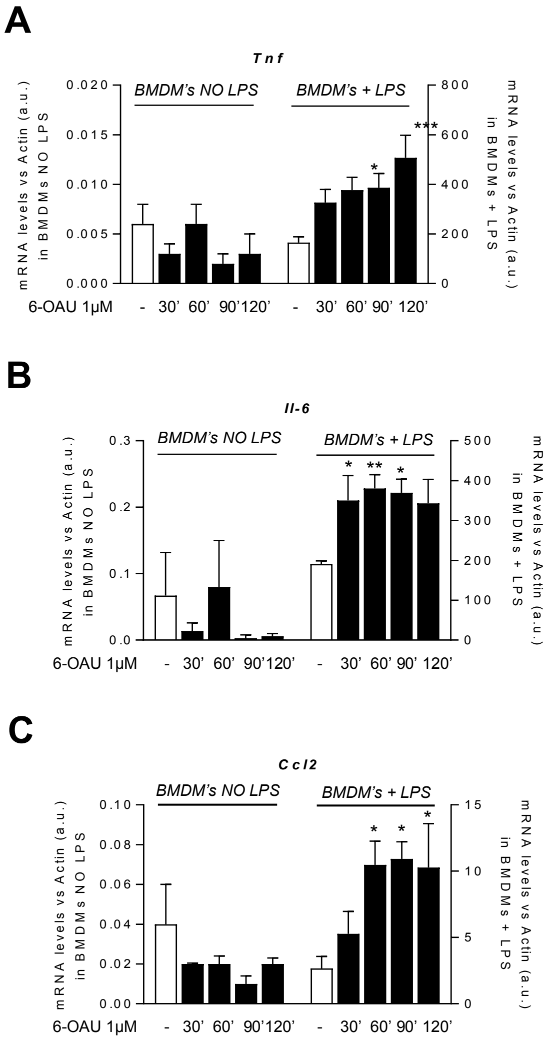

Supplementary Figure 5. 6-OAU does not significantly stimulate inflammatory gene expression in macrophages in a non-inflammatory context (i.e. no LPS pre-treatment).

Naïve BMDMs with no LPS pre-treatment (left Y axis **A**, **B**, **C**) and BMDMs pre-treated with LPS (0.1 µg/ml) for 2 hours (right Y axis **A**, **B**, **C**) were stimulated with either vehicle (0.3% DMSO) or 1 µM 6-OAU for 30, 60, 90, and 120 minutes. mRNA expression of *Tnfα* (**A**), *Il-6* (**B**), and *Ccl2* (**C**) was analysed by q-PCR. Data presented as mean ± S.E.M of n = 3-6 separate experiments. Statistical significance was assessed using one-way ANOVA with Dunnett´s multiple comparison posthoc test. * $P \leq 0.05$, **$P \leq 0.01$, ***$P \leq 0.001$ versus vehicle.

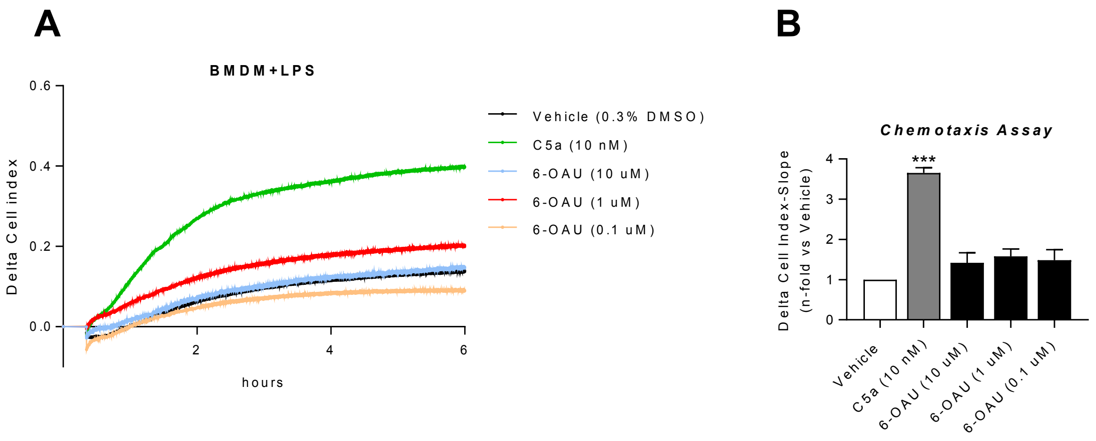

Supplementary Figure 6. 6-OAU is not a chemoattractant compound for BMDMs.

BMDMs were treated with LPS (0.1 µg/ml) for 2 hours prior to being placed into the upper chamber of a CIM-16 plate in a 16 well E-plate of an ACEA xCELLigence RTCA-DP machine (100.000 cells/well). Chemotaxis was measured for 6 hours at 37°C towards vehicle (0.3% DMSO), 10 nM C5a or different concentrations of 6-OAU (0.1 µM- 10 µM). (**A**) Response curves of migration cell index are shown and are representative from n=3 independent experiments. (**B**) Quantification of BMDM chemotaxis by Δ CI analysis. Data are mean + SEM of n=3 separate experiments with 2 technical replicates per condition. ***P≤0.001 versus vehicle.

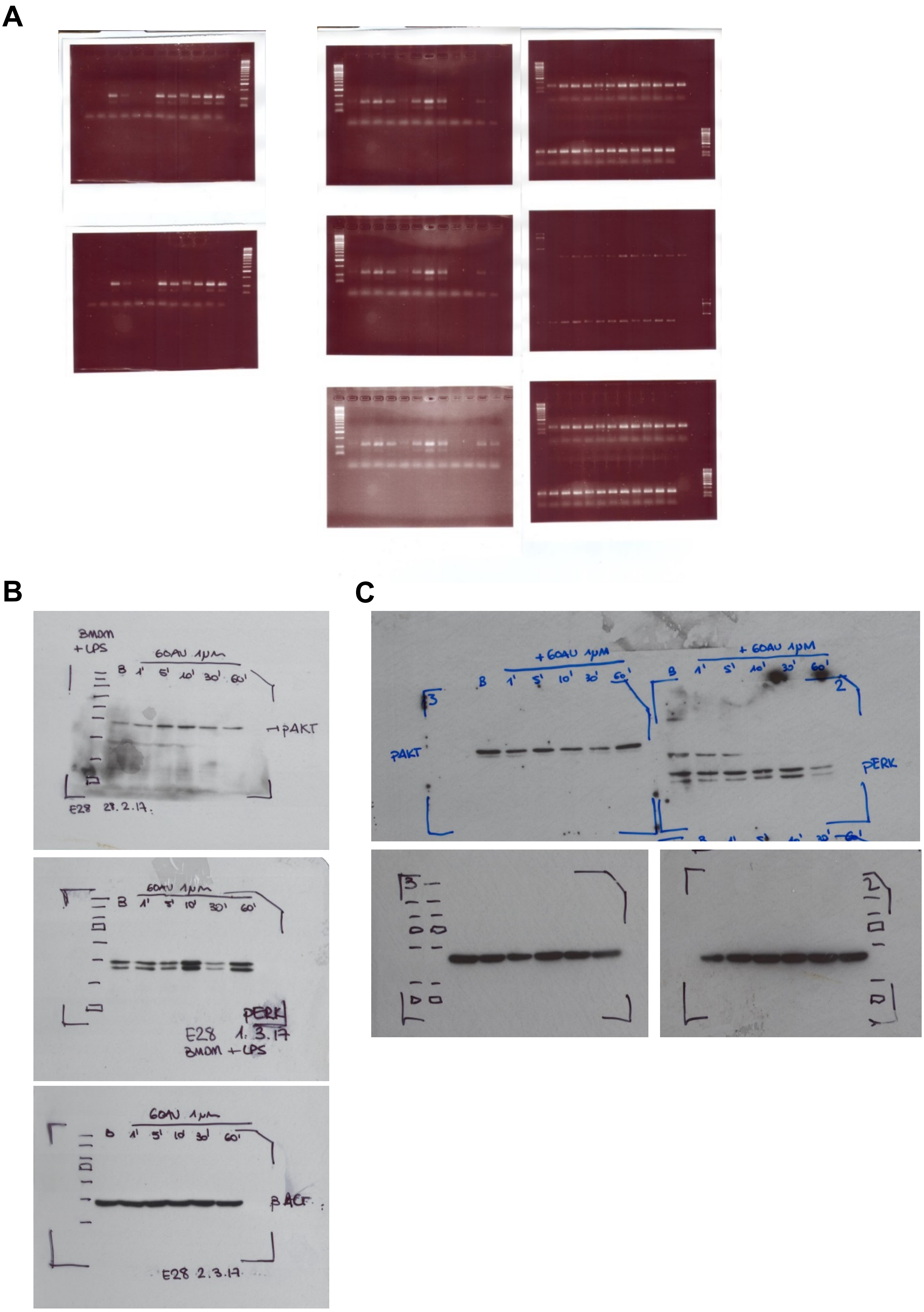
A
B
C
BMDM +LPS
6OAU 1µM
pAKT
E28 28.2.17
pERK
E28 1.3.17
BMDM +LPS
βACT
E28 2.3.17
+ 6OAU 1µM
PERK

D

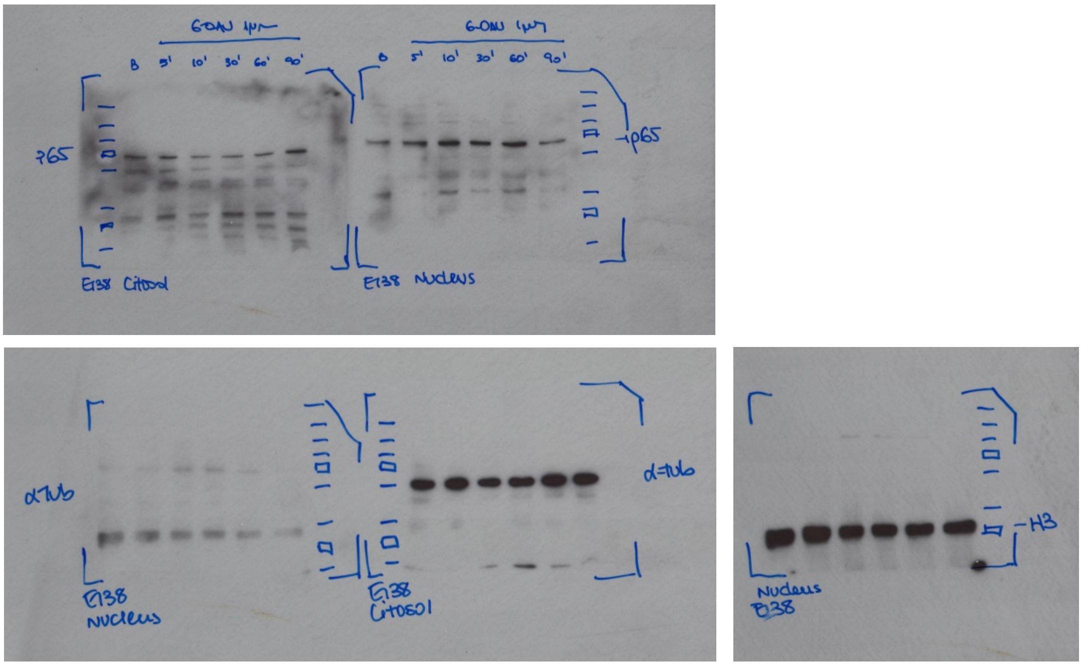

E

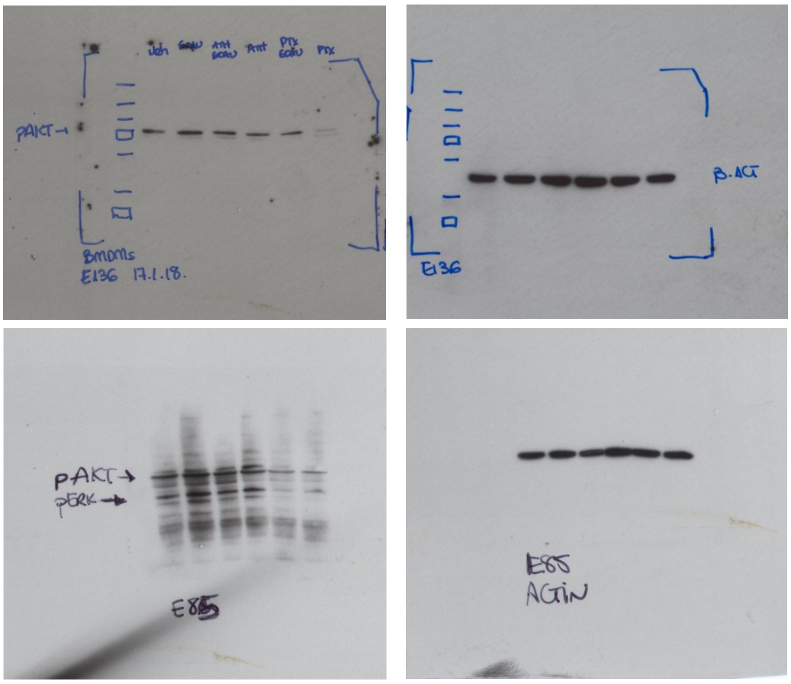

F

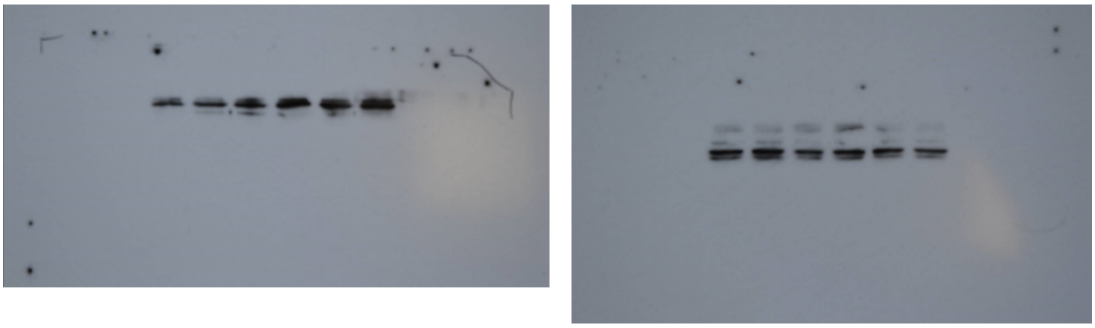

Supplementary Figure 7. Full, uncropped gels and western blots.

Full, uncropped agarose gels of RT-PCR experiments from figure 1C (**A**), and uncropped western blots corresponding to figure 5A (**B, C**), to figure 5C (**D**), and to figure 7B (**E**) are shown. Uncropped western blots corresponding to same experiments as those shown in figure 5A but using total ERK and total Akt as loading controls (**F**).

Materials and Methods

GPR84 Antagonist synthesis

Synthesis of GPR84 antagonist:

General methods.

All commercially available reagents and solvents were used without further purification unless specified. H_2O was de-ionised and microfiltered using a Milli-Q® Millipore machine. Brine refers to a sat. aq. solution of sodium chloride. *In vacuo* refers to the use of a rotary evaporator attached to a diaphragm pump. All reactions using water-sensitive reagents were carried out under an N_2 atmosphere and using oven dried glassware. Thin layer chromatography was performed on aluminium plates coated with 60 F254 silica and visualised under UV light (254 nm). Column chromatography was carried out either using Kiesel gel 60 silica in a glass column, or using a Biotage SP4 automated flash column chromatography platform. Melting points were recorded on an EZ-Melt Automated Melting Point Apparatus (EZ Melt). Infrared spectra were recorded on a Bruker Tensor 27 FT-IR spectrometer as neat or thin film samples and selected characteristic peaks are reported in cm^{-1}. NMR spectra were recorded on Bruker Avance spectrometers (AVIII 400, AVII 500) in the deuterated solvent stated, with chemical shifts (δ) reported in ppm. The multiplicity of each signal is indicated by: s (singlet); br. s (broad singlet); d (doublet); t (triplet); q (quartet); dd (doublet of doublets); td (triplet of doublets); qt (quartet of triplets); or m (multiplet). Accurate mass measurements were obtained to four decimal places using a Bruker MicroTOF spectrometer by the mass spectrometry service of the Chemistry Research Laboratory, University of Oxford, UK. Low resolution mass spectra and LC/MS spectra were obtained using an Agilent 1260 Infinity II with Diode Array and Single Quadrupole Detectors. The LCMS method was as follows: Agilent Poroshell 120 EC-C18 2.7 µm, 50 x 4.6 mm; A = water + 0.1% formic acid; B = MeCN + 0.1% formic acid; 40 °C; %B: 0.00 min 20%, 4 min 98%, 6 min 98%, 6.10 min 20%, 9.95 min 20%; 0.9 mL/min.

Synthetic scheme:

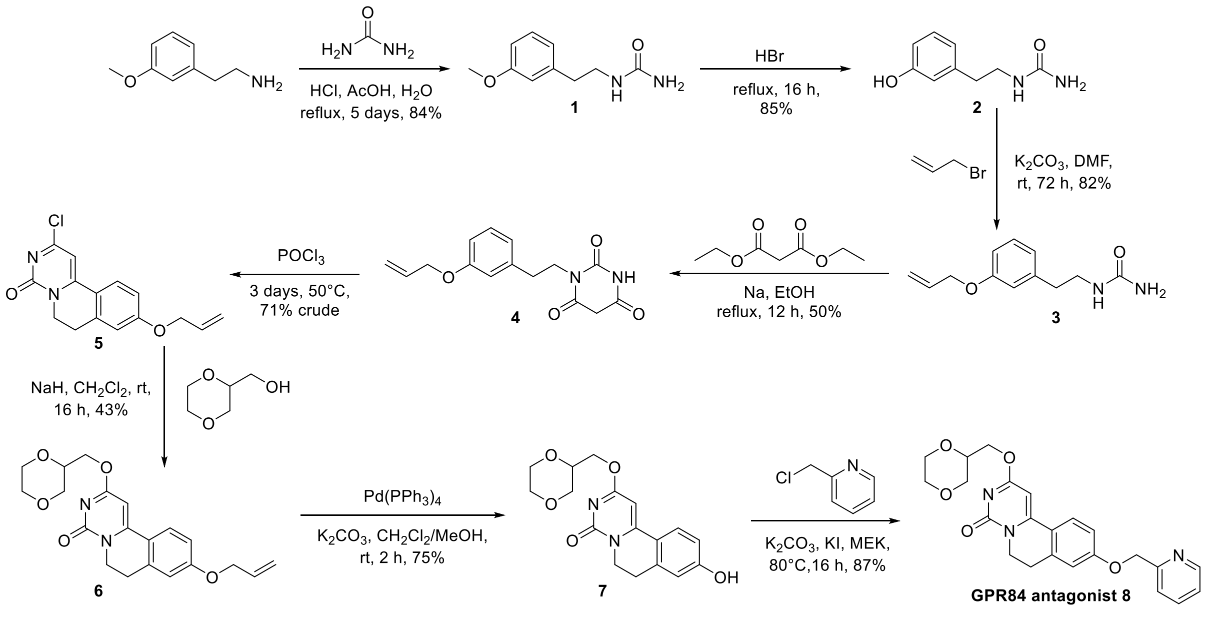

1-(3-Methoxyphenethyl)urea (1)

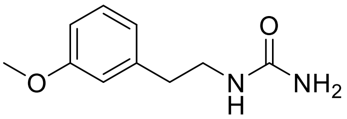

3-Methoxyphenethylamine (8.63 g, 57.1 mmol) and urea (13.7 g, 228 mmol) were dissolved in H_2O (69 ml) before sequential addition of conc. HCl (1.04 mL) and AcOH (3.1 ml), and the reaction stirred at reflux for 5 d. After cooling to rt, the formed solid was filtered, washed with H_2O, and dried to afford **1** as an off-white solid (9.37 g, 84%); **mp** 99–101 °C (H_2O); **ν_{max}** (thin film) 3452 (N–H), 3332 (N–H), 2956 (C–H), 1644 (C=O), 1586, 1553; **^{1}H NMR** (400 MHz, $CDCl_3$) δ 7.21 (1H, app t, J = 7.7 Hz), 6.83–6.68 (3H, m), 4.85 (1H, br t, J = 5.8 Hz), 4.49 (2H, br s), 3.73 (3H, s), 3.40 (2H, td, J = 6.8, 5.8 Hz), 2.77 (2H, t, J = 6.8 Hz); **^{13}C NMR** (101 MHz, $CDCl_3$) δ 159.8, 158.7, 140.7, 129.6, 121.2, 114.6, 111.8, 55.2, 41.6, 36.2; **LRMS (ESI$^+$)** 195 $[M+H]^+$; **HRMS (ESI$^+$)** $C_{10}H_{14}N_2O_2$ $[M+H]^+$ *calc.* 195.1128, *found* 195.1128.

1-(3-Hydroxyphenethyl)urea (2)

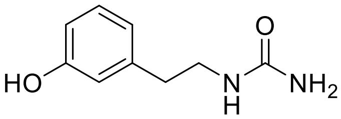

A solution of **1** (9.00 g, 46.3 mmol) in conc. HBr (63 mL, 48 % w/w aq.) was stirred at reflux for 20 h. The mixture was cooled, basified with NaOH (5M, aq.), and extracted with EtOAc (4 × 50 mL). The organic phases were dried ($MgSO_4$), filtered, and concentrated *in vacuo* to give **2** as a grey solid (7.12 g, 85%); **mp** 115–118 °C (EtOAc); **ν_{max}** (thin film) 3439 (N–H), 3333 (N–H), br 3013 (O–H), 1637 (C=O), 1581, 1537; **^{1}H NMR** (400 MHz, DMSO-d_6) δ 9.27 (1H, br s), 7.06 (1H, app t, J = 7.2 Hz), 6.63–6.56 (3H, m), 5.91 (1H, t, J = 6.0 Hz), 5.45 (2H, s), 3.16 (2H, td, J = 7.5, 6.0 Hz), 2.57 (2H, t, J = 7.5 Hz) ; **^{13}C NMR** (101 MHz, DMSO-d_6) δ 158.1, 157.8, 141.6, 129.7, 119.7, 116.0, 113.4, 41.2, 36.7; **LRMS (ESI$^+$)** 181 $[M+H]^+$; **HRMS (ESI$^+$)** $C_9H_{12}N_2O_2$ $[M+H]^+$ *calc.* 181.0972, *found* 181.0970.

1-(3-(Allyloxy)phenethyl)urea (3)

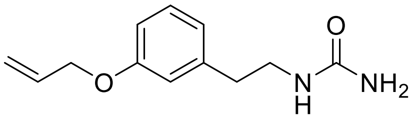

Allyl bromide (4.03 g, 33.3 mmol) was added to a solution of **2** (3.00 g, 16.7 mmol) and K_2CO_3 (6.90 g, 49.9 mmol) in DMF (20.7 mL) and stirred at rt for 3 d. The mixture was evaporated to dryness and the crude residue taken up in EtOAc (100 mL). The organic phase was washed with H_2O (60 mL), aq. sat. Na_2CO_3 (60 mL), and brine (60 mL), dried ($MgSO_4$), filtered, and concentrated *in vacuo.* The crude product was purified using silica gel column chromatography (95:5 CH_2Cl_2/MeOH) to afford **3** as a white solid (3.01 g, 82%); **mp** 75–77 °C (MeOH); **ν_{max}** (thin film) 3359 (N–H), 3158 (N–H), 2947 (C–H), 1678 (C=C), 1637 (C=O), 1593; **^{1}H NMR**

(400 MHz, CD_3OD) δ 7.15 (1H, t, J = 8.1 Hz), 6.81–6.69 (3H, m), 6.02 (1H, ddt, J = 17.3, 10.4, 5.2 Hz), 4.48 (2H, dt, J = 5.2, 1.6, 1.6 Hz), 3.29 (2H, t, J = 7.3 Hz), 2.7 (2H, t, J = 7.3 Hz); **^{13}C NMR** (101 MHz, CD_3OD) δ 160.8, 158.8, 140.9, 133.6, 129.1, 121.0, 116.0, 114.9, 112.2, 68.3, 41.1, 36.0; **LRMS (ESI$^+$)** 221 $[M+H]^+$; **HRMS (ESI$^+$)** $C_{12}H_{16}N_2O_2$ $[M+H]^+$ *calc.* 221.1285, *found* 221.1285.

1-(3-(Allyloxy)phenethyl)pyrimidine-2,4,6(1*H*,3*H*,5*H*)-trione (4)

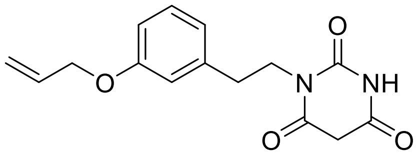

Sodium metal (209 mg, 9.08 mmol) was dissolved in EtOH (14.6 mL), before addition of diethyl malonate (1.45 g, 9.08 mmol) and the mixture heated at reflux for 1 h. Compound **3** (1.00 g, 4.54 mmol) was then added and the mixture further heated at reflux for 12 h. The reaction was cooled to rt and acidified with HCl (1M, aq.). The resulting precipitate was filtered, washed with H_2O and dried *in vacuo* to afford **4** as a white solid (1.30 g, 50%); **mp** 149–151 °C (H_2O); **ν_{max}** (thin film) 3193, 3091, 2964 (C–H), 1708 (C=O), 1681 (C=O), 1662 (C=O); **^{1}H NMR** (400 MHz, $CDCl_3$) δ 8.26 (1H, s), 7.21 (1H, app t, J = 7.8 Hz), 6.86–6.77 (3H, m), 6.05 (1H, ddt, J = 17.2, 10.6, 5.3 Hz), 5.42 (1H, dq, J = 17.2, 1.5 Hz), 5.29 (1H, dq, J = 10.6, 1.5 Hz), 4.53 (2H, dt, J = 5.3, 1.5 Hz), 4.12–4.04 (2H, m), 3.62 (2H, s), 2.92–2.83 (2H, m); **^{13}C NMR** (101 MHz, $CDCl_3$) δ 165.0, 164.4, 158.9, 150.3, 139.2, 133.4, 129.7, 121.6, 117.8, 115.6, 113.1, 68.9, 42.5, 39.4, 34.1; **LRMS (ESI$^+$)** 289 $[M+H]^+$; **HRMS (ESI$^+$)** $C_{15}H_{16}N_2O_4$ $[M+H]^+$ *calc.* 289.1183, *found* 289.1183.

9-(Allyloxy)-2-chloro-6,7-dihydro-4H-pyrimido[6,1-a]isoquinolin-4-one (5)

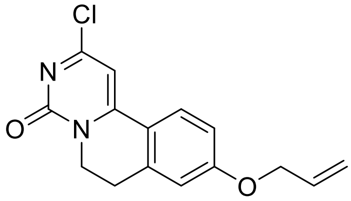

A solution of **4** (1.10 g, 3.82 mmol) in $POCl_3$ (8.25 mL) was heated at 50 °C for 3 d. After cooling, $POCl_3$ was evaporated, the crude residue taken up in CH_2Cl_2 (60 ml) and quenched with aq. sat. $NaHCO_3$ (30 mL). The organic phase was further washed with aq. sat. $NaHCO_3$ (30 mL), H_2O (2 × 30 mL), and brine (30 mL). The organic phase was dried ($MgSO_4$), filtered, and concentrated *in vacuo*. Alkene **5** was obtained as an orange solid (782 mg, crude yield 71%) was used in the next step without further purification.

2-((1,4-Dioxan-2-yl)methoxy)-9-hydroxy-6,7-dihydro-4H-pyrimido[6,1-a]isoquinolin4-one (6)

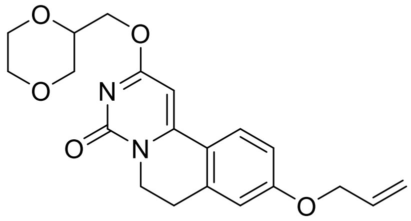

(1,4-Dioxan-2-yl)methanol (363 mg, 3.08 mmol) was added to a suspension of NaH (123 mg, 3.08 mmol) in CH_2Cl_2 (1.9 mL) at 0 °C and stirred at rt for 15 min before addition of a solution of compound **5** (444 mg, 1.54 mmol) in CH_2Cl_2 (4.5 mL). The mixture was warmed to rt and stirred for 16 h. The reaction was quenched with aq. sat. NH_4Cl (5 mL) and the organic phase washed with H_2O (5 mL), dried ($MgSO_4$), filtered, and concentrated *in vacuo* to give **6** as a pale yellow solid (242 mg, 43%); **mp** 141–142 °C (CH_2Cl_2); **ν_{max}** (thin film) 2955, 2852, 1651 (C=O), 1606; **1H NMR** (400 MHz, $CDCl_3$) δ 7.63 (1H, d, J = 8.8 Hz), 6.91 (1H, dd, J = 8.8, 2.6 Hz), 6.80 (1H, d, J = 2.6 Hz), 6.28 (1H, s), 6.05 (1H, ddt, J = 17.2, 10.5, 5.3 Hz), 5.43 (1H, dq, J = 17.2, 1.5 Hz), 5.34 (1H, dq, J = 10.5, 1.5 Hz), 4.61 (2H, dt, J = 5.3, 1.5 Hz), 4.48–4.32 (2H, m), 4.19 (2H, t, J = 6.5 Hz), 3.97 (1H, dddd, J = 10.0, 6.4, 3.8, 2.6 Hz), 3.89–3.69 (4H, m), 3.65 (1H, ddd, J = 11.6, 10.6, 3.0 Hz), 3.48 (1H, dd, J = 11.5, 10.0 Hz), 2.97 (2H, t, J = 6.5 Hz); **^{13}C NMR** (101 MHz, $CDCl_3$) δ 170.8, 161.7, 157.2, 152.6, 138.4, 132.4, 127.9, 120.0, 118.5, 114.7, 113.9, 89.2, 73.4, 69.1, 67.9, 66.8, 66.5, 66.1, 40.5, 28.3; **LRMS (ESI$^+$)** 371 $[M+H]^+$; **HRMS (ESI$^+$)** $C_{20}H_{22}N_2O_5$ $[M+H]^+$ *calc.* 371.1602, *found* 371.1599.

2-((1,4-Dioxan-2-yl)methoxy)-9-hydroxy-6,7-dihydro-4H-pyrimido[6,1-a]isoquinolin4-one (7)

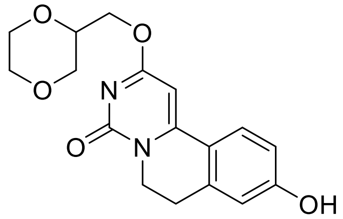

K_2CO_3 (44.7 mg, 324 mmol), $Pd(PPh_3)_4$ (9.36 mg, 8.10 µmol), and **6** (60.0 mg, 162 µmol) were dissolved in degassed CH_2Cl_2/MeOH (1:1, 1.6 mL). The mixture was further degassed with N_2 and stirred at rt for 2 h before addition of H_2O (2 mL) and the aqueous layer separated. The pH was adjusted to pH 1 with HCl (2M, aq.) and the resulting precipitate was filtered, washed with H_2O and dried to afford **7** as a pale yellow solid (40 mg, 75%); **mp** 254–256 °C (H_2O); **ν_{max}** (thin film) 3013 (O–H), 2957, 1637 (C=O); **1H NMR** (400 MHz, DMSO–d_6) δ 10.31 (1H, s), 7.84 (1H, d, J = 8.6 Hz), 6.77 (1H, dd, J = 8.6, 2.5 Hz), 6.74 (1H, d, J = 2.5 Hz), 6.45 (1H, s), 4.29–4.19 (2H, m), 3.98 (2H, t, J = 6.5 Hz), 3.84 (1H, dddd, J = 10.0, 6.0, 4.0, 2.6 Hz) 3.80–3.73 (2H, m), 3.70-3.63 (1H, m), 3.60 (1H, app td, J = 11.2, 2.6 Hz), 3.49 (1H, app td, J = 11.1, 2.6 Hz), 3.37 (1H, dd, J = 11.4, 10.0 Hz), 2.90 (2H, t, J = 6.5 Hz); **^{13}C NMR** (125 MHz, DMSO–d_6) δ 170.7, 161.4, 156.2, 153.3, 139.2, 129.1, 118.3, 115.4, 114.7, 87.8, 73.3, 67.6, 66.2, 66.2, 65.6, 40.2*, 27.5**;** **LRMS (ESI$^+$)** 331 $[M+H]^+$; **HRMS (ESI$^+$)** $C_{17}H_{18}N_2O_5$ $[M+H]^+$ *calc.* 331.1289, *found* 331.1287. *obscured under solvent peak - visible by HSQC.

2-((1,4-Dioxan-2-yl)methoxy)-9-hydroxy-6,7-dihydro-4H-pyrimido[6,1-a]isoquinolin-4-one (GPR84 antagonist 8)

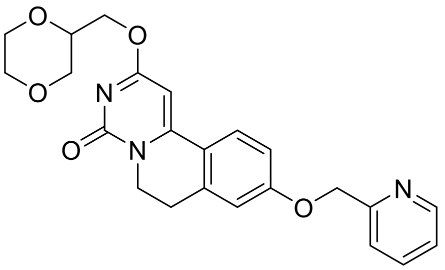

A solution of **7** (30 mg, 90.8 µmol), 2-(chloromethyl)pyridine (22.3 mg, 136 µmol), K_2CO_3 (43.9 mg, 318 µmol), and KI (15.1 mg, 90.8 µmol) in MEK (1.5 ml) was heated at 80 °C for 16 h in a sealed tube. After cooling, the mixture was evaporated to dryness and the crude residue purified by silica gel column chromatography (97:3 CH_2Cl_2/MeOH) to give **GPR84 antagonist 8** as a white solid (33 mg, 87%); **mp** 196– 198 °C (MeOH); **ν_{max}** (thin film) 2955, 1662 (C=O), 1602, 1593; **^{1}H NMR** (400 MHz, $CDCl_3$) δ 8.62 (1H, d, *J* = 4.9 Hz), 7.74 (1H, app td, *J* = 7.7, 1.8 Hz), 7.63 (1H, d, *J* = 8.8 Hz), 7.49 (1H, d , *J* = 7.9 Hz), 7.29–7.23 (1H, m), 6.98 (1H, dd, *J* = 8.8, 2.6 Hz), 6.88 (1H, d, *J* = 2.6 Hz), 6.27 (1H, s), 5.26 (2H, s), 4.46–4.35 (2H, m), 4.19 (2H, t, J = 6.5 Hz), 3.97 (1H, dddd, *J* = 10.0, 6.3, 3.8, 2.6 Hz), 3.88-3.70 (4H, m), 3.65 (1H, ddd, J = 11.7, 10.7, 3.1 Hz), 3.48 (1H, dd, *J* = 11.5, 10.1 Hz), 2.96 (2H, t, *J* = 6.5 Hz); **^{13}C NMR** (101 MHz, $CDCl_3$) δ 170.8, 161.4, 157.2, 156.3, 152.5, 149.6, 138.5, 137.1, 128.0, 123.1, 121.5, 120.4, 114.8, 114.1, 89.3, 73.4, 71.0, 67.9, 66.8, 66.5, 66.1, 40.5, 28.3; **LRMS (ESI$^+$)** 444 $[M+Na]^+$; **HRMS (ESI$^+$)** $C_{23}H_{23}N_3O_5$ $[M+H]^+$ *calc.* 422.1711, *found* 422.1700.

LCMS trace for GPR84 antagonist 8

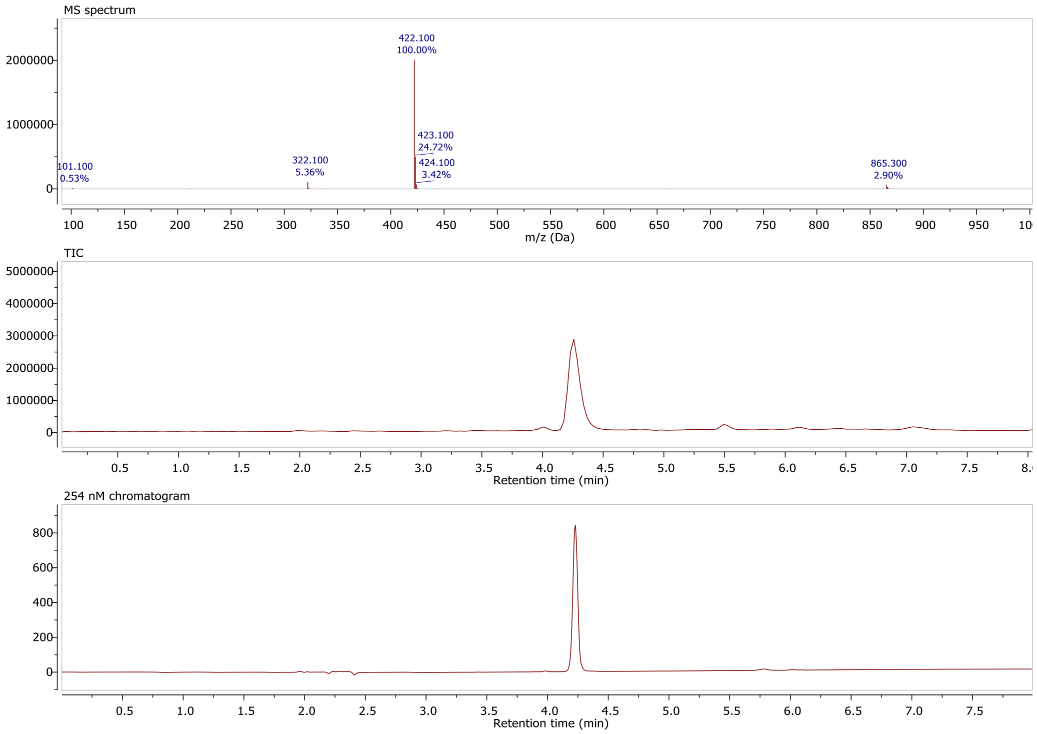

^{1}H NMR for GPR84 antagonist 8

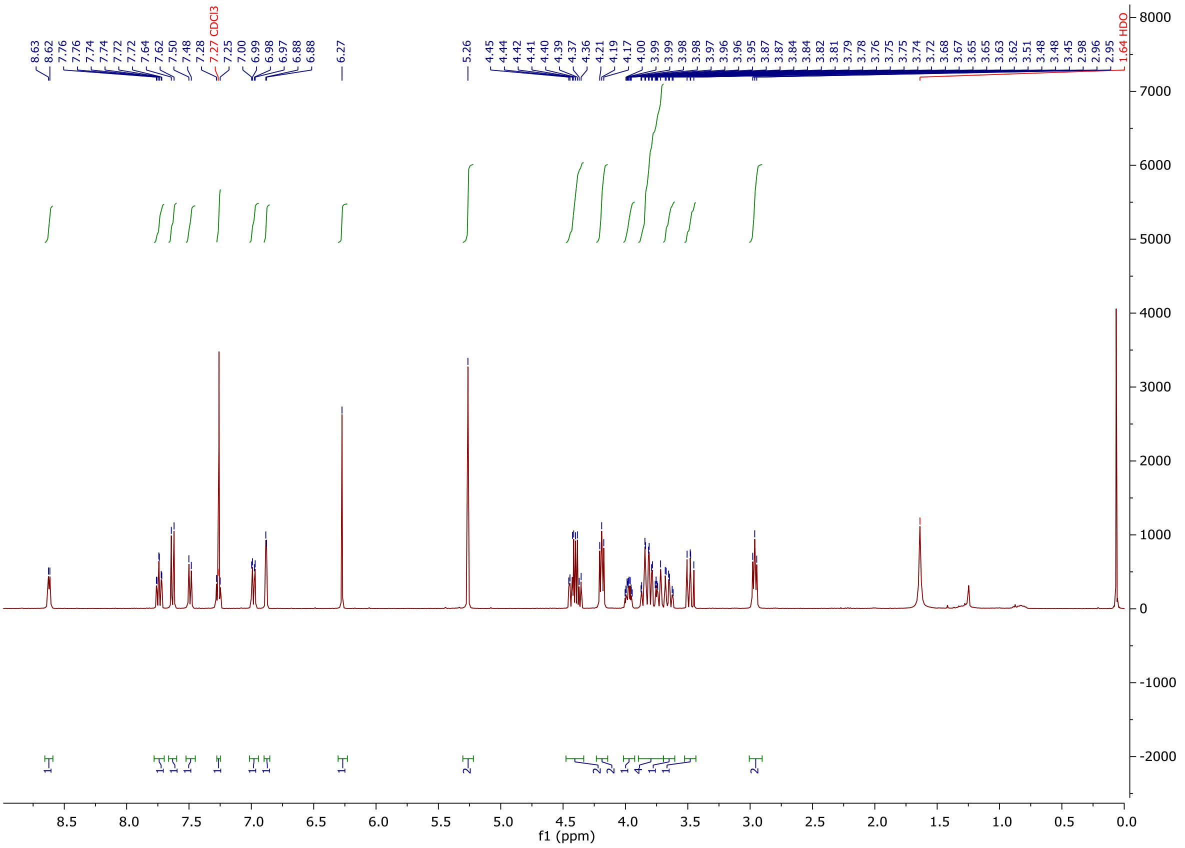

^{13}C NMR for GPR84 antagonist 8

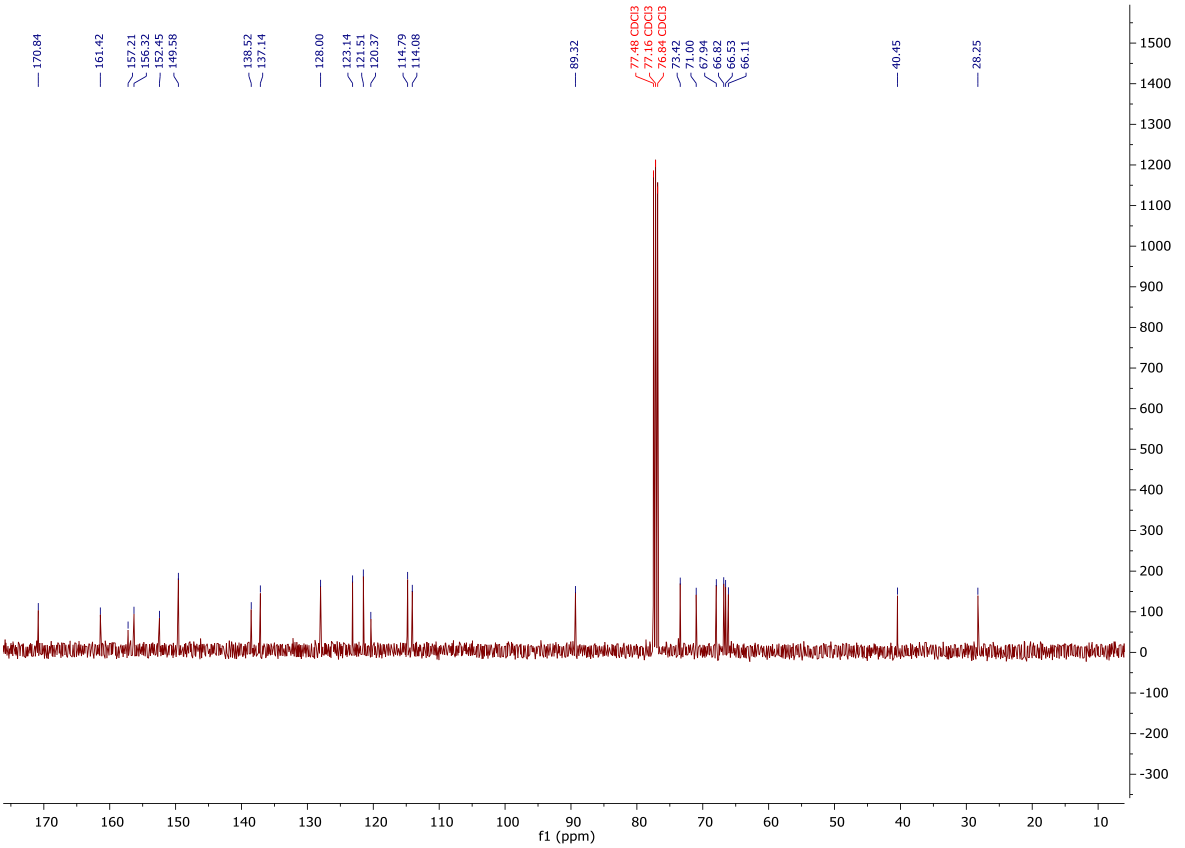

Supplement: Supplementary file 1 [file data_sheet_1.PDF]
